# Navigating the chaos: a scoping review of gaps in disaster nursing and a roadmap for the future

**DOI:** 10.1186/s12912-025-04088-4

**Published:** 2025-11-13

**Authors:** Amir Khorram-Manesh, Zakaria Mani

**Affiliations:** 1 Department of Surgery, Institute of Clinical Sciences, Sahlgrenska Academy, University of Gothenburg, Gothenburg, Sweden; 2 Disaster Medicine Center, University of Gothenburg, Gothenburg, Sweden; 3 Gothenburg Emergency Medicine Research Group (GEMREG), Sahlgrenska University Hospital, Gothenburg, Sweden; 4 Nursing Department, Jazan University, Jazan, Saudi Arabia

**Keywords:** Disaster, Education, Emergency, Nursing, Psychology, Roles

## Abstract

**Introduction:**

The increasing frequency and complexity of global disasters demand a highly competent and resilient nursing workforce. While nurses are essential across all phases of disaster management, the specialty of disaster nursing faces systemic challenges that undermine its effectiveness. This scoping review synthesizes academic literature to identify and address critical gaps within the field.

**Method:**

A structured scoping review methodology was employed, analyzing peer-reviewed articles to identify recurring themes and deficiencies.

**Results and findings:**

The findings highlight significant shortcomings in five key areas: (1) inadequate and inconsistent education and training; (2) ambiguous roles, responsibilities, and scopes of practice; (3) the profound and often unaddressed psychological toll on nurses; (4) nascent evidence base due to a lack of robust research; and (5) systemic logistical and organizational barriers. These gaps compromise patient outcomes, responder safety, and the overall resilience of the healthcare system in crises.

**Conclusion:**

Based on this analysis, a series of evidence-informed recommendations are proposed to advance the specialty. These include developing a standardized educational framework, formalizing disaster nursing roles through policy and credentialing, integrating comprehensive mental health support systems, promoting a targeted research agenda, and strengthening interprofessional collaboration with nurses in leadership roles. Addressing these critical areas is paramount to building a sustainable and effective disaster nursing workforce capable of meeting the challenges of the 21st century.

**Clinical trial number:**

Not applicable

**Supplementary Information:**

The online version contains supplementary material available at 10.1186/s12912-025-04088-4.

## Introduction

The contemporary global landscape is marked by a notable increase in the frequency and intensity of disasters. This escalation is driven by a complex interplay of factors, including climate change, geopolitical instability, rapid urbanization, and the emergence of novel pandemics. Together, these converging forces have transformed mass casualty incidents from a peripheral concern to a central challenge for global health systems [1, 2]. According to data from the Center for Research on the Epidemiology of Disasters (CRED) and the United Nations Office for Disaster Risk Reduction (UNDRR), the global burden is substantial. In 2022 alone, 387 natural hazard-induced disasters led to over 30,700 deaths, affected 185 million individuals, and caused an estimated $223.8 billion in economic losses [3]. 

This trend is amplified in humanitarian crises driven by conflict, climate change, and economic instability. Reports such as the International Rescue Committee’s (IRC) “Emergency Watchlist 2024” highlight numerous escalating crises where converging shocks disproportionately affect vulnerable populations, overwhelming local health systems and creating unprecedented demand for humanitarian aid (Table 1) [4–14]. Beyond these immediate causes, the report highlights four underlying systemic issues that perpetuate these emergencies: (1) A global preference for conflict over diplomacy, (2) The increasing targeting of civilians in warfare, (3) The disproportionate impact of climate change on vulnerable countries, and (4) Growing economic inequality worldwide. In response, the IRC calls for urgent and sustained international action. Key recommendations include increased funding for climate adaptation efforts and the development of a more effective humanitarian aid system [4, 15]. 

**Table 1:** Summarizes the top 10 humanitarian crises from the IRC’s *Emergency watchlist 2024* report [4–14]

Country/reference	Crisis Description
Sudan [4, 5]	Devastating war causing massive displacement and near-collapse of humanitarian aid.
Occupied Palestinian Territory (Gaza) [4–6]	Catastrophic crisis due to intensified hostilities and lack of essential resources.
South Sudan [4–7]	Critical food insecurity worsened by conflict, economic crisis, and flooding.
Burkina Faso [4–8]	Armed group violence limiting access to essential services.
Myanmar [4–9]	Military takeover leading to conflict, displacement, and restricted aid.
Mal [4–10]	UN peacekeeping withdrawal increasing violence and complicating aid delivery.
Somalia [4–11]	Climate shocks (drought and flooding) driving humanitarian needs.
Niger [4–12]	Coup causing political/economic instability and reduced public service capacity.
Ethiopia [4–13]	Complex crisis from drought, conflict, and inflation.
Democratic Republic of Congo [4–14]	Escalating conflict in the east worsening a long-standing humanitarian crisis.

Adding to these crises, widespread wildfires, and catastrophic flooding have made mass casualty incidents a central concern for global health systems [16, 17]. Within this volatile context, nurses, as the largest group of healthcare professionals, are indispensable frontline responders. Their roles span the entire disaster management cycle, from mitigation and preparedness to response and recovery [18]. They are responsible for providing direct patient care, performing triage, managing public health initiatives, offering psychosocial support, and coordinating care in challenging and chaotic environments.

### A brief history of disaster nursing

The formalization of disaster nursing as a specialty evolved from the pioneering field work of figures like Florence Nightingale and Clara Barton to a structured discipline in the 20th century, accelerated by global conflicts and large-scale public health crises [19, 20]. Key events like 9/11 and Hurricane Katrina exposed critical gaps in preparedness, leading to the creation of structured response frameworks and international standards, culminating in the ICN’s 2019 Framework of Disaster Nursing Competencies [21]. However, despite the establishment of these international standards, a significant gap persists between defining *what* competencies nurses should have and *how* to operationally and institutionally ensure they are prepared, supported, and able to implement them effectively [22, 23]. 

Disaster nursing has emerged as a vital and recognized specialty that encompasses all phases of the disaster cycle: mitigation, preparedness, response, and recovery. Nevertheless, despite its critical importance, the field continues to face persistent challenges in institutional support, education, and global workforce preparedness [22, 23]. The gap between competency frameworks and practical implementation has been a central theme in recent research.

A comprehensive 2021 overview by Hugelius synthesized the field’s progress, concluding that while research has successfully identified core competencies, significant knowledge gaps remain [24]. The literature is heavily skewed toward acute-phase hospital response and self-perceived competencies, with far less known about long-term recovery roles or the efficacy of different educational models. The author specifically called for further investigation into the organizational, systemic, and educational factors that enable or inhibit the translation of competencies into practice. This mapping of the research frontier highlights that the field must now move beyond *what* nurses should do and explore *how* health systems can practicably train, deploy, and support them, particularly in low-resource and high-stress contexts.

To date, this call for research into the organizational and systemic prerequisites for a prepared nursing workforce remains largely unanswered. While frameworks *exist*, we know little about how nursing leadership structures, institutional resource allocation, and specific educational interventions actually impact nurse preparedness and response efficacy in real-world mass casualty incidents.

### Aims

This paper aims to address this critical gap. to provide a comprehensive scoping review of the current literature to identify the primary gaps and shortcomings in disaster nursing. This review was guided by the following research questions:

1. What are the primary documented gaps and shortcomings in disaster nursing education, practice, policy, and research?
2. What are the recurring recommendations in the literature to address these identified gaps?

By synthesizing existing knowledge, this analysis seeks to inform a strategic discussion and propose actionable recommendations.

## Methodology

### Study design

From May 30 to June 30, 2025 data were collected to explore the challenges and gaps in disaster nursing. A scoping literature review was conducted, grounded in a systematic and transparent search methodology, following the guidelines provided by Arksey and O’Malley [25], further refined by Levac et al. [26] and guided by the PRISMA-ScR checklist (Supplementary file 1) [27]. This structured approach, while not adhering to the strict protocol of a systematic review, was crucial for providing a robust foundation for this study. The review aimed to synthesize existing knowledge by first systematically identifying relevant literature and then organizing the findings into a cohesive narrative that highlights key themes and areas for improvement. While a formal quality appraisal of each included study is not a mandatory step in the Arksey and O’Malley framework [25], this review ensured a baseline level of quality by limiting inclusion to peer-reviewed research, systematic reviews, and meta-analyses, as detailed in the exclusion criteria.

### Searching keywords

A comprehensive list of keywords and Medical Subject Headings (MeSH) terms was used to conduct the literature search. Core search terms included: “disaster nursing,” “disaster preparedness,” “emergency nursing,” “mass casualty incident,” and “nurses.” These primary terms were strategically chosen to capture the core concepts of the research topic. To narrow and refine the search, these terms were combined with secondary terms, which helped to identify specific aspects of challenges and gaps. The secondary terms included: “gaps,” “shortcomings,” “challenges,” “competencies,” “education,” “scope of practice,” “psychological impact,” “mental health,” and “research.” This approach ensured a multi-faceted search that went beyond general disaster nursing literature to specifically target the issues and areas for improvement within the field.

### Search strings

The keywords were combined using Boolean operators to create comprehensive search strings, which were adapted to the syntax of each database. The search strategy involved multiple combinations of core terms (e.g., ‘disaster nursing’) with various secondary terms (e.g., ‘education’, ‘challenges’, ‘scope of practice’). For example, one of the primary search strings used was: *((“disaster nursing” OR “emergency nursing” OR “mass casualty incident”) AND (“gaps” OR “shortcomings” OR “challenges” OR “barriers”)) AND (“education” OR “competenc*” OR “scope of practice” OR “psychological” OR “mental health”). This iterative approach allowed for a targeted yet extensive search. The final search string was: (“disaster nursing” OR “mass casualty incident”) AND (“gaps” OR “shortcomings” OR “challenges”). This method allowed for a targeted yet extensive search of the databases.

### Search databases

The literature search was conducted across several major electronic databases to ensure a broad coverage of academic literature. The selected databases were PubMed, CINAHL (Cumulative Index to Nursing and Allied Health Literature), Web of Science, and Scopus. These sources were chosen for their extensive coverage of medical, nursing, and interdisciplinary health sciences literature, providing a wide array of peer-reviewed articles. PubMed and CINAHL are particularly strong in the health and nursing fields, while Scopus and WoS offer a broader, more interdisciplinary reach, helping to capture relevant articles from various academic disciplines.

### Eligibility, inclusion and exclusion criteria

To ensure the relevance and quality of the selected literature, specific inclusion and exclusion criteria were established:

#### Inclusion criteria

Peer-reviewed research articles, systematic reviews, and meta-analyses to ensure that only high-quality, scientifically sound literature was included in the review. Articles published in the English language was a practical necessity to ensure accurately interpreting and analyzing the content. Articles published between 2010 and 2025 were chosen to ensure the findings were relevant to the contemporary disaster landscape, reflecting recent advancements, challenges, and research trends in the field.

#### Exclusion criteria

Non-peer-reviewed literature (i.e., editorials and opinion pieces), as well as articles where nursing wasn’t the primary focus and studies that concentrated on single, localized events without offering broader insights into the field, were excluded to maintain the scientific rigor of the review. While recognizing the value of grey literature (e.g., government and NGO reports), the focus of this review was to synthesize the state of the *academic* and *peer-reviewed* evidence base to identify gaps in formal research. This ensured that our review was based on high-quality, relevant research that contributed to a general understanding of the challenges and gaps in disaster nursing.

### Study selection

Titles and abstracts were screened for relevance based on the predefined inclusion and exclusions criteria. The full texts of the selected articles were retrieved for eligibility. Efforts were made to minimize biases by applying consistent criteria and documenting decisions transparently. Both authors agreed on the included studies.

### Data charting, analysis, collation, summary and reporting results

Data extraction was conducted manually using a structured form designed to capture key information from each article. This included the title, authors, year of publication, country of origin, study design, key findings, and specific details regarding the identified and recommendations. This systematic approach ensured comprehensive data collection for subsequent analysis.

To synthesize the findings, a thematic analysis was performed using NVivo—one of the most widely used qualitative data analysis (QDA) software tools [28]. NVivo facilitates the organization, storage, and analysis of data from diverse sources such as interviews, surveys, and academic articles. It is particularly effective for thematic synthesis, allowing users to create “nodes” to represent themes, code text to these nodes, and visualize relationships between concepts. To minimize bias, a structure coding process was employed. An initial codebook was developed based on the data extraction form and the review’s research questions. This codebook was iteratively refined as data was coded. To ensure consistency (intra-coder reliability), a sample of articles (20%, *n* = 4) was re-coded after a two-week period to check for discrepancies. Themes were generated only after all articles were coded, and final themes were cross-checked against the original articles to ensure they were representative of the source material. The resulting themes were then consolidated into major categories, which are presented in the results section of the paper to provide a clear and structured overview of the findings.

## Results

**Fig. 1:**
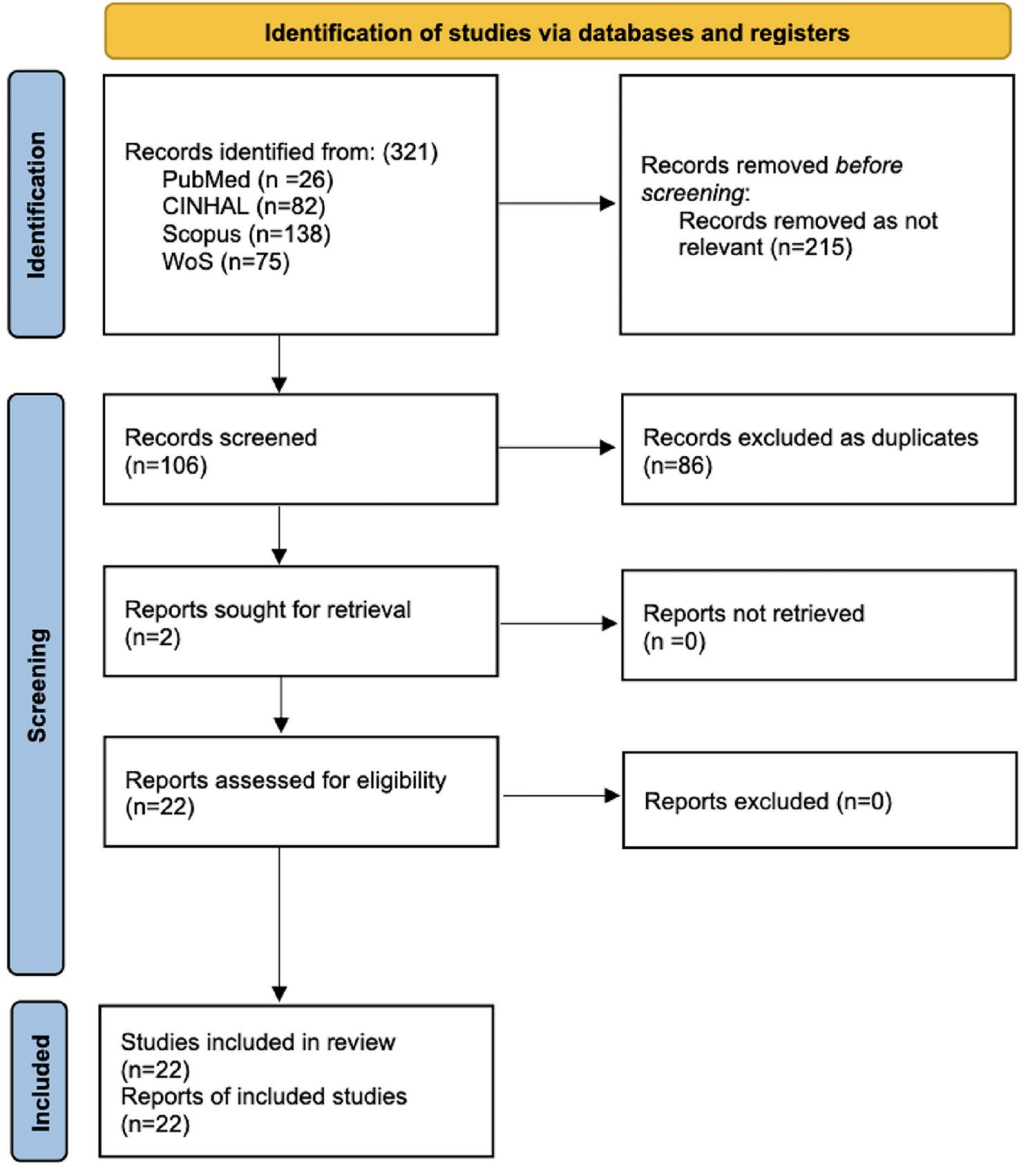
Shows the PRISMA flowchart diagram for this study

The initial search yielded a high number of articles. After filtering the outcomes by the inclusion criteria, 321 studies were qualified for initial review (PubMed 26; Scopus 138; WoS 75; CINAHL 82). Titles and abstracts were screened for relevance, reducing the number to 106 articles for full-text review (215 not relevant). From this pool, 20 key articles explicitly addressed systemic gaps, challenges, or recommendations for disaster nursing and were selected for final inclusion. Two more studies from the list of included studies were found eligible for inclusion. In total, 22 articles were included in this study (Fig. 1, and supplementary file 2), providing a comprehensive overview of the current state of disaster nursing research. Published between 2015 and 2025, they represent a recent and relevant body of evidence, featuring a variety of research methodologies, including scoping reviews, systematic reviews, cross-sectional surveys, and descriptive correlational studies, reflecting a multifaceted approach to investigating the complexities of disaster nursing. The research spans diverse geographical settings, from the Asia-Pacific region to a global context and addresses a wide spectrum of themes. Key areas of focus include disaster nursing competencies, the psychological impact of disasters on nurses, gaps in education and training, and the need for stronger leadership roles for nurses in disaster response. Collectively, these studies highlight the challenges and opportunities in the field and provide a foundation for evidence-based recommendations to strengthen disaster nursing practice, education, and policy. Summary of each included study is illustrated in Table 2.

**Table 2:** The summary and characteristics of all included studies

No.	Title	Journal, Date, Author (ref)	Study Setting	Study Outcomes	Comments
1	Emergency healthcare providers’ perceptions of preparedness and willingness to work during disasters and public health emergencies.	Healthcare (MDPI), 2020, Sultan et al. [23]	Response, perception, education, training	Emergency healthcare providers generally recognize the importance of preparedness for disasters and public health emergencies. There is variability in perceived preparedness levels among providers. Willingness to work during such events is influenced by factors such as: Availability of personal protective equipment (PPE), Institutional support, Training and clear communication, Concerns about personal safety and family responsibilities can reduce willingness to work.	The findings highlight a critical gap between perceived preparedness and actual readiness. While providers acknowledge the need for preparedness, systemic issues—such as inadequate resources and unclear protocols—impact their confidence and willingness to respond. This suggests that healthcare systems need to invest in Comprehensive disaster training programs, Adequate resource allocation (especially PPE), Psychological support for staff during crises
2	Disaster nursing research: A scoping review of the nature, content, and trends of studies published during 2011–2020	Int Emerg Nurs, 2021, Hugelius [24]	Scoping Review	The two most common research topics were: Competencies: Assessing nurses’ self-perceived skills, educational needs, and readiness for disasters. Psychological Impact: Describing the stress, anxiety, PTSD, and coping mechanisms of nurses after a disaster. Most studies were qualitative or descriptive (e.g., surveys, interviews). Research was heavily concentrated, with most publications coming from the USA, Australia, and Iran.	There is a major lack of studies that test solutions (e.g., “Does this specific training program *actually* improve preparedness?” “Does this support system *actually* reduce PTSD?“). Research overwhelmingly ignores the mitigation and long-term recovery phases of the disaster cycle. There is a critical shortage of research from low- and middle-income countries (LMICs) and other highly disaster-prone regions.
3	Cross-sectional survey of the disaster preparedness of nurses across the Asia-Pacific region.	Nurs Health Sci, 2015, Usher et al. [28]	Healthcare system in Asia-Pacific region	Acknowledged gap in nursing curricula; focus on Asia-Pacific nurses’ perceptions; low-to-moderate levels of preparedness; individual factors are most significant; policy and education implications.	Provides regional, data-driven evidence of a global problem and suggests that individual-level interventions are key to improving preparedness.
4	Development of Disaster Nursing Education and Training Programs (2000–2019): A systematic review.	Systematic review, Nurse Educ Today, 2021, Loke et al. [29]	Systematic review Global Education and Training Programs	Growth and global inequities in education; imbalance in educational content; diverse and modern teaching approaches; positive learning outcomes; call for further research and broader scope.	Points out the progress and gaps in disaster nursing education, noting the need for more comprehensive curricula and equitable access.
5	Disaster Nursing for Early Career Emergency Nurses.	Review, J Emerg Nurs, 2025, Rizek J. [30]	Review, Academic Nursing Programs and Emergency Nursing	Foundational skills for emergency nurses; focus on standardization in academic programs; broad scope of disaster nursing; importance of personal qualities; contribution to community and global health.	Emphasizes that disaster nursing is a specialized extension of an emergency nurse’s core skills and highlights the need for a more prepared workforce.
6	Nurses as Leaders in Disaster Preparedness and Response- A call to action.	J Nurs Scolar, 2016, Veenema et al. [31]	Healthcare leadership	A vision for disaster nursing’s future; collaborative and expert-driven methodology; comprehensive recommendations; leadership roles for nurses; building national resilience.	Presents a forward-looking, consensus-based approach to improving the field, advocating for an expanded leadership role for nurses.
7	Building health care system capacity: Training Health Professionals in Disaster Preparedness Coalitions.	Prehosp Disaster Med, 2015, Walsh et al. [32]	Healthcare coalitions training and education	Focus on learning from established leaders; key training needs identified; major challenges to training; the value of an online resource repository; success of mature coalitions.	Identifies both the strengths of HCCs and the systemic barriers to effective training, proposing a practical solution for resource sharing.
8	Could the Internet of Things be used to enhance student nurses’ experience in a disaster simulation	Observational, Online J Nurs Inform, 2018, Lapante et al. [33]	Nursing education simulation	Context of disaster nursing education; simulation as a key teaching method; proposal for integrating the Internet of Things (IoT); nursing’s slow adoption of IoT; a framework for a meaningful introduction of IoT.	Presents an innovative idea for modernizing disaster nursing education by using technology to create more realistic and engaging simulations.
9	Disaster Nursing Competencies in a time of Global Conflicts and Climate Crises.	Cross-sctional survey, Int Nurs Rev, 2025, Ličen and Prosen. [34]	Response and education	There is a critical need to integrate disaster nursing education into both undergraduate and continuing professional development programs. Policy makers and healthcare institutions must prioritize disaster preparedness to ensure effective nursing response during crises. The study advocates for international collaboration to develop and implement competency frameworks that are adaptable across different regions and disaster contexts.	The study highlights a concerning gap in disaster nursing competencies, especially in practical and psychosocial skills. Nurses with formal training and prior experience showed better preparedness, emphasizing the need for structured education. The lack of standardized global frameworks and limited integration of disaster topics in nursing curricula point to systemic issues. Overall, the findings call for urgent policy and educational reforms to strengthen nursing readiness in the face of growing global crises.
10	Nurses’ disaster preparedness and core competencies in Turkey: a descriptive correlational design.	Descriptive, Int Nurs Rev, 2019, Taskiran and Baykal. [35]	Preparedness, education	Turkish nurses acknowledge the importance of disaster preparedness, but their actual preparedness levels are moderate to low. There are notable gaps in disaster-related training and education, particularly in practical response skills and coordination. A positive correlation was found between nurses perceived competencies and their level of preparedness. Experience and prior training significantly influenced preparedness levels.	The study calls for **e**nhanced disaster nursing education in both undergraduate programs and ongoing professional development. It recommends **s**ystematic integration of disaster preparedness into nursing curricula and institutional policies. Strengthening core competencies through simulation, drills, and interdisciplinary collaboration is essential for improving disaster response capacity.
11	Common challenges in the prehospital management of mass-casualty incidents: A systematic integrative review	Systematic integrative review, Prehosp Disaster Med, 2024, Hugelius and Becker. [36]	Prehospital MCI Management	Limited evidence on best practices; focus on common challenges; importance of strategy and improvisation; need for specialized training; situational awareness as a core competency.	Underscores the critical need for research and highlights that a successful response requires a balance between structured plans and adaptability.
12	Challenges for nurses in disaster management: A scoping review.	Scoping review, Risk Manag Healthc Policy, 2020, Al Harthi et al. [37]	Disaster response settings	Identification of key barriers; disaster nursing as a “new” specialty; use of a scoping review methodology; focus on ethical and legal issues; call to action for stakeholders.	Provides a clear, categorized list of obstacles and argues that many of the problems stem from the field’s nascent stage of development.
13	Nurses’ Roles in Nursing Disaster Model: A Systematic Scoping Review.	Scoping review, Iran J Public Health, 2021, Firouzkouhi et al. [38]	Scoping review	The review identified the crucial roles of nurses in all three phases of a disaster: before, during, and after. These roles include preparing for the disaster, providing care and managing emergencies during the event, and assisting with evacuation and rehabilitation afterward.	Highlights a lack of clear, universally mandated roles for nurses during disasters, despite their critical importance, necessitating a need for more education and training in disaster nursing, as well as the development of national guidelines and protocols to better define their responsibilities.
14	Nurses’ experience of ethical preparedness for public health emergencies and healthcare disasters: A systematic review of qualitative evidence.	Systematic review, Nurs Health Sci, 2014, Johnstone and Turale. [39]	Systematic review, Public health emergencies and disasters.	A significant gap in research; focus on qualitative research; confirmed lack of data; the need for focused attention; informing the ethical basis of management.	Highlights a critical and under-researched aspect of disaster nursing, arguing that ethical considerations must be more intentionally integrated into all phases of disaster management.
15	Why a disaster is not just normal business ramped up: Disaster response among ED Nurses.	Qualitative Study, Australasian Emerg Care, 2018, Hammad et al. [40]	Emergency departments	A disaster changes the familiar ED environment; a chain reaction of changes; initial shock and altered behavior; the reality of disaster response; recommendations for enhanced training.	Emphasizes that disaster response is fundamentally different from a nurse’s everyday work and requires specialized, reality-based training.
16	Overcoming challenges in nursing disaster preparedness and response: an umbrella review.	Review, BMC Nurs, 2024, Al Thobaity. [41]	Healthcare Systems	Comprehensive nature of challenges; use of a rigorous review methodology; identification of nine key thematic strategies; emphasis on education, technology, and collaboration; ultimate goal of improving patient outcomes.	Demonstrates that disaster nursing faces multifaceted problems and proposes a structured framework for overcoming them with evidence-based strategies.
17	The psychological impact of quarantine and how to reduce it: Rapid review of the evidence.	Review, The lancet, 2020, Brooks et al. [42]	Rapid review of infectious outbreak	The review found that quarantine often has negative psychological effects, including post-traumatic stress symptoms, confusion, and anger. The primary factors contributing to these effects were found to be quarantine duration, fear of infection, frustration, boredom, inadequate supplies or information, financial loss, and social stigma.	The study suggests that to reduce the psychological burden of quarantine, officials should keep it as short as possible, provide a clear rationale and adequate information, and ensure people have sufficient supplies. It also highlights the value of promoting an altruistic mindset—reminding people that their sacrifice helps protect the wider community.
18	Disaster nursing: A retrospective review.	Review, Crit Care Nurs Clin North Am, 2010, Stangeland. [43]	Disaster Response	A gap in research on nurses’ perspectives; nurses as both responders and affected individuals; the need for research to inform policy; the importance of understanding “intent to respond”; broader implications for education, practice, and policy.	Highlights a crucial void in the literature, focusing on the personal experiences of nurses and how these insights can lead to better policies.
19	Telenursing in Incidents and Disasters: A systematic review.	Systematic Review, J Emerg Nurs, 2020, Nejadshafiee et al. [44]	Remote disaster care (Telehealth)	Technology as a solution to healthcare shortages; focus on telehealth and telenursing; three main findings; a gap in “telenursing care” research; the promise of telenursing.	Dentifies a critical gap in research and argues that telenursing offers a viable solution to the shortage of healthcare professionals in disaster areas.
20	Learning from Terrorist Mass Casualty Incidents: a global survey.	Mix-method study, BJ Anaesth, 2022, Tallach et al. [45]	International healthcare systems	Focus on human factors over physical supplies; key challenges: communication, security, and blast injuries; importance of re-triage and coordination; the value of flexibility and preparedness exercises; persisting challenges: training and psychological well-being.	Reframes disaster response as a human and organizational challenge rather than a logistical one, providing practical insights for improvement.
21	A literature review on the impact of disasters on healthcare systems, the role of nursing in disaster management, and strategies for cancer care delivery in disaster-affected populations.	Review, Front Oncol, 2023, Wang et al. [46]	Disaster Settings & Tumor Management	Intersectional focus on disaster nursing and oncology; crucial role of nurses across all phases of disaster; use of case studies and successful examples; addressing challenges and opportunities; guidance for future research and practice.	Highlights a unique and often overlooked area of disaster healthcare, showing the versatility and specialization of a nurse’s role.
22	History of Disaster Nursing: From Nightingale to 21st Century	Qualitative interpretive review, J Res Nurs, 2022, Fletcher et al. [47]	Global Healthcare and nursing	Historical importance and global role of nurses; key themes from the historical review; evolution beyond traditional nursing roles; advancements and remaining gaps; comprehensive scope of disaster nursing.	Provides a historical context for the field, showing its evolution from an instinctual “sense of duty” to a formalized, strategic discipline.

The thematic analysis of the literature revealed five consistent and interconnected areas of deficiency, gaps and shortcomings in disaster nursing.

### Inadequate and inconsistent education and training

A predominant theme is the absence of standardized, competency-based disaster education within academic and professional nursing programs. Much of the available training is voluntary and ad hoc, leading to significant gaps in preparedness [24, 29]. Even when programs exist, they often prioritize theory over the practical skills essential for disaster response [24, 30]. This deficit is particularly acute for early-career nurses who lack experiential knowledge [31]. Studies, such as those by Sultan et al., highlight that this stems from hierarchical issues, where disaster preparedness is not mandated or integrated from a policy level into core curricula [23]. Consequently, nurses lack proficiency in critical competencies like mass-casualty triage, resource allocation, and, most fundamentally, situational awareness (SA) and effective decision-making needed to operate in chaotic environments [24, 32]. While solutions like interdisciplinary training coalitions [33] and advanced simulations [34] show promise, they are not yet the standard [35, 36]. 

### Ambiguity in roles and responsibilities

In the chaos of a disaster, the lack of clearly defined roles and legal protections for nurses creates a critical barrier to effective response. As historical reviews show, the nurse’s role in a crisis, while central, often remains informally defined [19]. This ambiguity fuels confusion in command structures [37] and forces nurses to operate in a gray zone, often expanding their scope of practice without clear guidelines or legal standing [38, 39]. For instance, a nurse may hesitate to perform a necessary life-saving procedure that is technically outside their day-to-day scope for fear of future liability, thereby delaying care. In another case, a response team’s efforts may be paralyzed by role confusion when multiple nurses with different specializations arrive on a scene with no clear leader designated. This confusion cripples a team’s collective SA and prevents the implementation of coherent disaster management strategies. The problem is magnified by the underutilization of nurses in leadership positions where their skills would be invaluable [31] and the profound ethical conflicts that arise from making high-stakes decisions in a vacuum [40]. 

### The unaddressed psychological toll on nurses

The immense psychological burden on nurses is a critical but often under-addressed issue. A disaster is not merely a busier shift; it is a fundamentally different context that inflicts extreme stress and moral distress [41]. Nurses are confronted with profound ethical dilemmas, such as rationing care, while simultaneously fearing for their own and their families’ safety [38, 40]. This is vividly illustrated in prolonged crises and conflict zones like Ukraine or Palestine, where nurses must contend not only with overwhelming casualties but also with the destruction of healthcare infrastructure and direct threats to their own lives [15]. The consistent failure of organizations to provide robust, accessible, and ongoing mental health support is a major systemic flaw [31, 42]. This neglect leads to high rates of burnout, PTSD (post-traumatic stress disorder), and moral injury, jeopardizing the long-term sustainability of the nursing workforce [43]. 

### Deficiencies in research and evidence-based practice

The challenges in education and practice are exacerbated by an underdeveloped evidence base for disaster nursing. The field’s reliance on systematic reviews signals an attempt to synthesize existing knowledge but also underscores the scarcity of primary research [24, 35–37, 42]. The reasons for this deficiency are twofold: first, the chaotic and unpredictable nature of disasters creates significant methodological barriers to conducting rigorous studies; second, there is a persistent lack of dedicated funding and institutional prioritization for this type of research. This gap forces a reliance on anecdotal experience rather than evidence-informed practice and impedes the development of validated educational competencies [30, 44], proven triage protocols, and effective strategies for managing disaster-specific injuries or implementing novel technologies like telenursing [29, 45]. 

### Logistical and organizational challenges

Individual competence is frequently nullified by systemic logistical and organizational failures. A consistent finding is the breakdown of core infrastructure during a response, including inter-agency communication [46], management of supplies [37], and the execution of inadequate surge plans [40, 41]. Crucially, these issues reveal a lack of dynamic organizational SA, where institutions fail to adapt plans in real-time to the evolving reality on the ground. This disconnect is often rooted in the exclusion of frontline nurses from high-level planning processes, resulting in strategies that are disconnected from the realities of care delivery [47]. Without robust and resilient systemic support, even the most skilled nurse cannot effectively deliver care, whether it’s for mass casualties or for vulnerable populations whose specialized supply chains are disrupted [48]. 

## Discussion

The collective findings from 22 included papers in this study paint a comprehensive picture of the current state of disaster nursing. While other reviews have focused on singular issues (e.g., education or mental health), the primary contribution of this study is its holistic synthesis of five interconnected areas of deficiency. It translates these findings into a ‘Roadmap for the Future’ (Table 3) to strengthen the specialty and enhance global health security. Despite progress, several critical and interconnected challenges persist. It’s clear that despite being the largest group of healthcare professionals, nurses aren’t adequately prepared for disasters. Their education often lacks standardized training in ethics, community health promotion, and all phases of disaster management—from mitigation to recovery. This creates a significant gap between their current skills and the demands of a crisis, forcing them to rely on improvisation over evidence-based practices [24, 29–48]. 

**Table 3:** A roadmap framework for advancing disaster nursing

Key Gap Identified	Actionable Recommendation	Primary Stakeholders
1. Inadequate & Inconsistent Education [29, 52–55]	Develop a global consensus framework for core competencies (based on ICN 2.0) and integrate it into all undergraduate nursing curricula.	Educational Institutions, WHO, ICN
	Mandate practical, skills-based training using high-fidelity simulation and interprofessional drills.	Healthcare Organizations, Universities
2. Ambiguity in Roles & Responsibilities [18, 21–23]	Lobby governments to establish clear legal frameworks defining scope of practice and providing liability protection during declared emergencies.	Professional Nursing Bodies, Government
	Develop and adopt a national/international credentialing system to validate specialized expertise and streamline deployment.	Professional Nursing Bodies
3. Unaddressed Psychological Toll [34–35, 37−38, 48, 50]	Implement a phased model of psychological support: pre-deployment resilience training, on-site support, and mandatory post-deployment screening.	Healthcare Organizations, Response Agencies
	Foster a culture of psychological safety through peer-support programs and leadership training.	Healthcare Leadership, Individuals
4. Deficiencies in Research [54, 56]	Establish and fund a dedicated research agenda for disaster nursing, prioritizing triage, mental health interventions, and health outcomes.	Funding Agencies, Research Institutions
	Foster international research collaborations to overcome methodological challenges and share data from diverse contexts.	Universities, NGOs
5. Logistical & Organizational Failures [34, 52]	Include nurses with disaster expertise in planning and policy-making bodies at all levels (hospital to national).	Government, Hospital Administration
	Empower nurses in leadership roles to ensure response plans are clinically realistic and effective.	Healthcare Organizations, Professional Bodies

The challenges nurses face goes beyond a simple knowledge gap. The field of disaster nursing is systemically undervalued, leading to a lack of defined roles and a fundamental ambiguity in their scope of practice [22, 24, 32, 33, 36, 49, 50]. This ambiguity complicates interprofessional collaboration and can create leadership vacuums during a crisis. It also places an undue burden on nurses, causing significant moral distress as they’re forced to act in roles for which they are not trained [32, 36, 38]. The personal toll of this work is immense; nurses report feelings of shock and disbelief, and their mental well-being is a looming public health crisis within the profession [43, 51–53]. A failure to address this through proactive mental health support is not only an ethical failure but a strategic one, as a burnt-out workforce cannot provide compassionate care and is prone to attrition.

To address these issues, research suggests several solutions [24, 30, 34, 54]. Technology like telehealth and telenursing can help manage staff shortages, while integrating tools like the Internet of Things (IoT) into simulations could make training more realistic [30, 34]. Fundamentally, nurses need to be more than just frontline responders; they must take on expanded roles as leaders, policymakers, and researchers [55]. Without a strong evidence base and a seat at the policy-making table, their vital contributions will continue to be marginalized, perpetuating a cycle where disaster plans lack the practical insights necessary for successful implementation. By investing in better training, supporting ethical research, and elevating nurses’ roles, we can build a more resilient healthcare system for future disasters [56–59]. 

It is crucial, however, to acknowledge the fundamental obstacles that prevent these gaps from being closed, particularly in low- and middle-income countries (LMICs). The recommendations for advanced simulation, robust mental health systems, and credentialing often presume a level of funding, infrastructure, and institutional stability that is non-existent in many settings identified by the IRC report [4]. Therefore, the primary barrier is often not a lack of *will* but a profound lack of *resources*. Implementing standardized education is difficult where basic nursing education is already strained, and promoting research is challenging where clinical capacity is overwhelmed. Future solutions must therefore be scalable, low-cost, and adaptable to low-resource settings to be globally effective [60].

## Recommendations for future progress

Addressing these deeply entrenched gaps requires a coordinated, multi-level strategy. The following “Roadmap Framework” (Table 3) synthesizes the key findings and proposes actionable recommendations targetedbat specific stakeholders to advance the field.

## Limitations

This review has several limitations that frame the findings and suggest areas for future research.

- **Scope of the Search**: The search strategy was focused, which introduces two limitations. First, it was limited to peer-reviewed articles from four databases (PubMed, CINAHL, WoS, and Scopus), potentially missing relevant articles in other databases. Second, it excluded “grey literature” (e.g., government/NGO reports, dissertations), which may omit practical, on-the-ground knowledge.
- **Language and Publication Bias**: A primary limitation is the English-language restriction. This approach inevitably skews the findings toward Anglophone, high-income nations and underrepresents the unique experiences and solutions from non-Anglophone regions in Africa, Asia, and Latin America. Furthermore, the review may be subject to publication bias, as studies with positive or statistically significant results are more likely to be published.
- **Interpretation and Synthesis**: The methodology has two dependencies. First, as a thematic analysis, although NVivo application was used, the findings involve researchers’ interpretation in coding and categorizing themes. Second, as a literature review, the study does not generate new empirical data. Its conclusions are entirely dependent on the quality and focus of the available primary studies, reflecting gaps *in the literature* rather than all gaps *in the field*.
- **Generalizability**: The review identifies broad, overarching themes. However, the primary research it is based on is often highly context-specific (e.g., hurricanes, pandemics). Care must be taken not to over-generalize these findings, as the challenges of a rapid-onset flood may differ significantly from those of a slow-onset famine or a prolonged conflict.

Despite these limitations, this review serves its intended purpose: to provide a robust synthesis of the dominant themes within the current academic discourse on disaster nursing. It successfully highlights critical, widely recognized areas for improvement and provides a solid foundation upon which future primary research, policy development, and educational reform can be built.

## Conclusion

Disasters are an inevitable feature of our world, but the challenges in our response are, however, addressable. Nurses are a cornerstone of disaster resilience, yet they are being asked to navigate the chaos without sufficient training, clear roles, psychological support, or an evidence-based playbook. The gaps identified in this review represent critical vulnerabilities in our global health security infrastructure. By systematically addressing these shortcomings through a concerted effort in education, policy, research, and leadership, we can empower the nursing profession to not only respond to disasters but to lead the way toward a safer and more resilient future. The time for incremental change has passed; a strategic and sustained investment in disaster nursing is a fundamental necessity.

## Supplementary Information

Below is the link to the electronic supplementary material.

Supplementary Material 1

Supplementary Material 2

## Data Availability

All data obtained has been provided and included in the manuscript. A summarized data extraction file is available as Additional file.

## Declarations

### Informed consents

Not applicable. This article does not contain any studies with human participants or animals by the authors, and no informed consent was required.

### AI

AI has not been used in the preparation of this manuscript.

## Abbreviations

SA: Situational Awarenes
CRED: Center for Research on the Epidemiology of Disasters
ICN: International Council of Nurses
IoT: Internet of Things
IRC: The International Rescue Committee’s
NGOs: Non-Governmental Organizations
PTSD: Post-Traumatic Stress Disorder
UNDRR: United Nations Office for Disaster Risk Reduction
WHO: World Health Organization
