# Supplementary material for: Navigating the chaos: a scoping review of gaps in disaster nursing and a roadmap for the future
Source: BMC Nurs. 2025 Nov 13;24:1396. doi: 10.1186/s12912-025-04088-4 (PMC12616989; doi:10.1186/s12912-025-04088-4)
Supplement: Supplementary file 2 — Supplementary Material 2 [file 12912_2025_4088_MOESM2_ESM.pdf]

Disaster Nursing August 2025, Revisited October 2025
PubMed= 26, included 6, duplicate 3, removed 17

A review of mass casualty incident triage tools for hospital-based triage.
Abdul-Nabi SS, Hitti E.Turk J Emerg Med. 2025 Oct 1;25(4):251-255. doi: 10.4103/tjem.tjem_77_25. eCollection 2025 Oct-Dec.PMID: 41104363 Free PMC article. Review.
Mass casualty incidents (MCIs) pose significant challenges to the healthcare systems, particularly in low-and lower-middle-income countries where prehospital triage is often limited, and hospitals face sudden surges of casualties. ...Existing research is limited by small s …

Disaster Preparedness and Response Among Healthcare Professionals During the Hajj: A Systematic Literature Review.
Alrabie T, Brown M, Rice B, Marsh L.Healthcare (Basel). 2025 Jun 30;13(13):1571. doi: 10.3390/healthcare13131571.PMID: 40648595 Free PMC article. Review.
A comprehensive database search was performed across CINAHL, Scopus, Medline, Embase, and APA PsycINFO, focusing on studies published between 2012 and 2025. Eligible studies addressed disaster nursing education, preparedness, and response. The Mixed Methods Appraisa …

Disaster Nursing for Early Career Emergency Nurses.
Rizek J.J Emerg Nurs. 2025 Jul;51(4):537-547. doi: 10.1016/j.jen.2025.03.020.PMID: 40602842 Review.
Recognizing its significance, disaster nursing competencies present an opportunity for further standardization within academic nursing programs. ...Additionally, the article highlights the importance of resilience, adaptability, and continuous learning as central to …
4

Common Challenges in the Prehospital Management of Mass-Casualty Incidents: A Systematic Integrative Review.
Hugelius K, Becker J.Prehosp Disaster Med. 2024 Aug;39(4):301-309. doi: 10.1017/S1049023X24000566. Epub 2024 Dec 12.PMID: 39663866
STUDY OBJECTIVE: This study aimed to analyze common challenges in prehospital MCI management. METHODS: Seventeen case studies or reports describing 15 MCIs (ie, terrorist attacks, chemical incidents, traffic accidents, weather-related incidents, and fires) were subject to …
5

Overcoming challenges in nursing disaster preparedness and response: an umbrella review.
Al Thobaity A.BMC Nurs. 2024 Aug 14;23(1):562. doi: 10.1186/s12912-024-02226-y.PMID: 39143575 Free PMC article. Review.
Disaster nursing plays a vital role in addressing the health needs of vulnerable populations affected by large scale emergencies. However, disaster nursing faces numerous challenges, including preparedness, logistics, education, ethics, recovery …
6

Family Members' Feedback on the "Quality of Death" of Adult Patients Who Died in Intensive Care Units and the Factors Affecting the Death Quality: A Systematic Review and Meta-Analysis.
Naya K, Sakuramoto H, Aikawa G, Ouchi A, Yoshihara S, Ota Y, Okamoto S, Fukushima A, Hirashima H.Cureus. 2024 Apr 15;16(4):e58344. doi: 10.7759/cureus.58344. eCollection 2024 Apr.PMID: 38756296 Free PMC article. Review.
The QODD-1 scale emerged as a frequently referenced and valuable metric for evaluating the quality of death in the ICU, and factors associated with the quality of ICU death were identified. However, research gaps persist, particularly regarding the variations in the qualit …

A literature review on the impact of disasters on healthcare systems, the role of nursing in disaster management, and strategies for cancer care delivery in disaster-affected populations.
Wang W, Li H, Huang M.Front Oncol. 2023 Jul 14;13:1178092. doi: 10.3389/fonc.2023.1178092. eCollection 2023.PMID: 37519811 Free PMC article. Review.
The article provides case studies and successful examples of nursing interventions in disaster settings and tumor management, emphasizing the challenges and opportunities in providing cancer care in disaster settings. Recommendations for future research and practice in …
8

"Learning From Disaster: What Past Events Can Teach Radiology Departments about Planning for a Mass Casualty Incident".
Hou C, Hafeez S, Okun J, Chaudhry H, Dym RJ.Curr Probl Diagn Radiol. 2023 Sep-Oct;52(5):418-424. doi: 10.1067/j.cpradiol.2023.05.009. Epub 2023 May 11.PMID: 37268454 Review.
The increased frequency of mass shootings, terror attacks, and natural disasters in recent years have presented challenges to provision of quality medical care in both short and long-term stressful situations. ...
9

Considerations of Medical Preparedness to Assess and Treat Various Populations During a Radiation Public Health Emergency.
Winters TA, Cassatt DR, Harrison-Peters JR, Hollingsworth BA, Rios CI, Satyamitra MM, Taliaferro LP, DiCarlo AL.Radiat Res. 2023 Mar 1;199(3):301-318. doi: 10.1667/RADE-22-00148.1.PMID: 36656560 Free PMC article. Review.
The inclusion of special populations in preclinical and clinical studies is essential to address shortcomings and is an important consideration for radiation public health emergency response planning. Pursuing this goal will benefit the population at large by considering t …
10

Education, training and technological innovation, key components of the ESTES-NIGHTINGALE project cooperation for Mass Casualty Incident preparedness in Europe.
Yánez Benítez C, Tilsed J, Weinstein ES, Caviglia M, Herman S, Montán C, Achatz G, Cuthbertson J, Ragazzoni L, Sdongos E, Ashkenazi I, Faccincani R.Eur J Trauma Emerg Surg. 2023 Apr;49(2):653-659. doi: 10.1007/s00068-022-02198-1. Epub 2022 Dec 13.PMID: 36513839 Free PMC article. Review.
This manuscript aims to describe the challenges of MCI triage, the education and training programs for MCI response in Europe, and the technological innovation that may aid optimal response. ...

Development of disaster nursing education and training programs in the past 20 years (2000-2019): A systematic review.
Loke AY, Guo C, Molassiotis A.Nurse Educ Today. 2021 Apr;99:104809. doi: 10.1016/j.nedt.2021.104809. Epub 2021 Feb 14.PMID: 33611142
With the call for all nurses to be prepared for disasters, disaster nursing education and training programs have expanded globally. However, a clear picture of the development and coverage of disaster nursing education and training programs is lacking. …
12

Transdisciplinary Research Priorities for Human and Planetary Health in the Context of the 2030 Agenda for Sustainable Development.
Ebi KL, Harris F, Sioen GB, Wannous C, Anyamba A, Bi P, Boeckmann M, Bowen K, Cissé G, Dasgupta P, Dida GO, Gasparatos A, Gatzweiler F, Javadi F, Kanbara S, Kone B, Maycock B, Morse A, Murakami T, Mustapha A, Pongsiri M, Suzán G, Watanabe C, Capon A.Int J Environ Res Public Health. 2020 Nov 30;17(23):8890. doi: 10.3390/ijerph17238890.PMID: 33265908 Free PMC article. Review.
It outlines a research agenda to address cross-cutting knowledge gaps to further understanding and management of the health risks of these global environmental changes through an expert consultation and review process. ...
13

Challenges for Nurses in Disaster Management: A Scoping Review.
Al Harthi M, Al Thobaity A, Al Ahmari W, Almalki M.Risk Manag Healthc Policy. 2020 Nov 16;13:2627-2634. doi: 10.2147/RMHP.S279513. eCollection 2020.PMID: 33235533 Free PMC article.
To reduce the impact of disasters, healthcare providers, especially nurses, need to be prepared to respond immediately. However, nurses face several challenges in all phases of disaster management. The findings of a literature review based on scoping approaches, which util …
14
Cite

Preparedness cycle to address transitions in diabetes care during the COVID-19 pandemic and future outbreaks.
Gujral UP, Johnson L, Nielsen J, Vellanki P, Haw JS, Davis GM, Weber MB, Pasquel FJ.BMJ Open Diabetes Res Care. 2020 Jul;8(1):e001520. doi: 10.1136/bmjdrc-2020-001520.PMID: 32690631 Free PMC article. Review.

The COVID-19 pandemic is considered a mass casualty incident of the most severe nature leading to unearthed uncertainties around management, prevention, and care. ...Implementing multidimensional frameworks may help identify gaps in care, alleviate ini …

15

Translating COVID-19 Pandemic Surge Theory to Practice in the Emergency Department: How to Expand Structure.

Paganini M, Conti A, Weinstein E, Della Corte F, Ragazzoni L.Disaster Med Public Health Prep. 2020 Aug;14(4):541-550. doi: 10.1017/dmp.2020.57. Epub 2020 Mar 27.PMID: 32216865 Free PMC article. Review.

The COVID-19 pandemic has presented the health-care system with challenges that have limited science to guide the staff, stuff, and structure surge response.This study reviewed the available surge science literature specifically to guide an emergency department's surge str …

16

The Challenges and Opportunities in Disaster Nursing Education in Turkey.

Kalanlar B.J Trauma Nurs. 2019 May/Jun;26(3):164-170. doi: 10.1097/JTN.0000000000000417.PMID: 31483775 Review.

The aim of this study is to assess the challenges and opportunities in disaster nursing education in Turkey. In this context, this article will discuss disaster nursing, the role of nurses in disasters, and topics related to disaster n …

17

Emergency Department Response to Chemical, Biological, Radiological, Nuclear, and Explosive Events: A Systematic Review.

Razak S, Hignett S, Barnes J.Prehosp Disaster Med. 2018 Oct;33(5):543-549. doi: 10.1017/S1049023X18000900.PMID: 30379127

Data were grouped into four themes: preparedness, response, decontamination, and personal protective equipment (PPE) problems.DiscussionThis study has recognized the ED as a system which depends on four key factors - preparedness, response, decontamination, and PPE problems - whi …

18

Antimicrobial Treatment for Systemic Anthrax: Analysis of Cases from 1945 to 2014 Identified Through a Systematic Literature Review.

Pillai SK, Huang E, Guarnizo JT, Hoyle JD, Katharios-Lanwermeyer S, Turski TK, Bower WA, Hendricks KA, Meaney-Delman D.Health Secur. 2015 Nov-Dec;13(6):355-64. doi: 10.1089/hs.2015.0033. Epub 2015 Dec 1.PMID: 26623698 Free PMC article.

Current national guidelines, developed for the individualized treatment of systemic anthrax, outline the use of combination intravenous antimicrobials for a minimum of 2 weeks, bactericidal and protein synthesis inhibitor antimicrobials for all cases of systemic anthrax, and at l …

19

Disaster nursing in Iran: challenges and opportunities.

Zarea K, Beiranvand S, Sheini-Jaberi P, Nikbakht-Nasrabadi A.Australas Emerg Nurs J. 2014 Nov;17(4):190-6. doi: 10.1016/j.aenj.2014.05.006. Epub 2014 Oct 24.PMID: 25440225 Review.

BACKGROUND AND OBJECTIVE: Disaster nursing was one of the first forms of nursing practice in Iran, and nurses have long served voluntarily in disasters. ...RESULTS: Analysis of the 32 articles yielded two major: organisational and managerial challenges, and …

20

Disaster preparedness and response: challenges for Australian public health nurses - a literature review.

Rokkas P, Cornell V, Steenkamp M.Nurs Health Sci. 2014 Mar;16(1):60-6. doi: 10.1111/nhs.12134.PMID: 24635900 Review.

This paper highlights issues currently facing disaster nursing and focuses on the challenges for Australian public health nurses responding to and preparing for disasters within Australia. ...

Preparedness and Response to a Rural Mass Casualty Incident: Workshop Summary.

Institute of Medicine (US) Forum on Medical and Public Health Preparedness for Catastrophic Events.Washington (DC): National Academies Press (US); 2011.PMID: 21977542 Free Books & Documents. Review.

Discuss opportunities to improve integration and coordination with public health systems to address challenges to national public health security, particularly in rural settings....

Disaster nursing: a retrospective review.
Stangeland PA.Crit Care Nurs Clin North Am. 2010 Dec;22(4):421-36. doi: 10.1016/j.ccell.2010.09.003.PMID: 21095551 Free PMC article. Review.
A plethora of information exists in the literature regarding emergencies and disasters. Nevertheless, significant gaps in the science related to nurses working during disasters are revealed. Few studies have addressed the perspective of nurses and their intent to respond t …
23

Pediatric and neonatal interfacility transport medicine after mass casualty incidents.
Lowe CG.J Trauma. 2009 Aug;67(2 Suppl):S168-71. doi: 10.1097/TA.0b013e3181af6086.PMID: 19667853 Review.
Transport teams are essential for the safe interfacility movement of critically ill patients.
A mass casualty incident (MCI) can present major challenges for a critical care transport team. ...
24

Disaster management teams.
Briggs SM.Curr Opin Crit Care. 2005 Dec;11(6):585-9. doi: 10.1097/01.ccx.0000186916.92757.ab.PMID: 16292064 Review.
A review of the current literature emphasizes the expanding role of disaster management teams as an integral part of the mass casualty incident response. RECENT FINDINGS: The incident command system has become the accepted standard for all disaster response. …
25

Practical considerations for providing pediatric care in a mass casualty incident.
Hohenhaus SM.Nurs Clin North Am. 2005 Sep;40(3):523-33, ix. doi: 10.1016/j.cnur.2005.04.014.PMID: 16111997 Review.
Plans for pediatric care during mass casualty incidents (MCIs) need to be developed. This article highlights challenges in providing care to children after MCIs and provides considerations for providing pediatric care....
26

Information technology and emergency medical care during disasters.
Chan TC, Killeen J, Griswold W, Lenert L.Acad Emerg Med. 2004 Nov;11(11):1229-36. doi: 10.1197/j.aem.2004.08.018.PMID: 15528589 Free article. Review.
Disaster response to mass-casualty incidents represents one of the greatest challenges to a community's emergency response system. Rescuers, field medical personnel, and regional emergency departments and hospitals must often provide care to large numbers …

Scopus
N=138, Included 13, duplicate 27, removed 98

Article • *Open access*
A silent epidemic: Exploring the clinico-epidemiological impact of explosion and gunshot injuries in the emergency department of a tertiary hospital in Somalia
Mohamed, H.H., Adan, H.A.A., Turfan, S., Aysin, M., Mohamud, M.F.Y.
African Journal of Emergency Medicine, 2025, 15(4), 100898

Article • *Open access*
Deployed in Disaster: Perspectives of Personnel Deployed in Ontario Long-Term Care During the Pandemic
Oldenburger, D., Baumann, A., Crea-Arsenio, M., Deber, R., Baba, V.
International Nursing Review, 2025, 72(3), e70083

Article • *Open access*
The Trinity Technique: A novel 3-step approach for debriefing interprofessional major incident simulation
Newton, J.
International Journal of Practice Based Learning in Health and Social Care, 2025, 13(1), pp. 26–41

Article
Disaster Nursing for Early Career Emergency Nurses
Rizek, J.
Journal of Emergency Nursing, 2025, 51(4), pp. 537–547

Article
Disaster nursing training needs among different levels of clinical nurses in China: A cross-sectional study with educational implications
He, J., Li, C., Zhou, T., ... Fu, Y., Tao, J.
Teaching and Learning in Nursing, 2025, 20(3), pp. e617–e622

Review • *Open access*
Disaster Preparedness and Response Among Healthcare Professionals During the Hajj: A Systematic Literature Review
Alrabie, T., Brown, M., Rice, B., Marsh, L.
Healthcare Switzerland, 2025, 13(13), 1571
1
Citations

Opens in a new tab.Entitled full textOpens in a new tab.

Article
Triage ethics in mass casualty incident simulation: A phenomenological exploration
Watson, A.L., Drake, J., Anderson, M., ... Reed, C., Rasmussen, R.
Nursing Ethics, 2025, 32(4), pp. 1313–1326

Article
Disaster Nursing Competencies in a Time of Global Conflicts and Climate Crises: A Cross-Sectional Survey Study
Ličen, S., Prosen, M.
International Nursing Review, 2025, 72(2), e70052

Article • *Open access*
Assessing the frontline competency gap: Emergency care perceptions among nurses in Yemen's conflict zone
Mani, Z.A., Innab, A., Taleb, F.
International Nursing Review, 2025, 72(2), e13047

Article • *Open access*
Unveiling the heart of disaster nursing: A qualitative study on motivations, challenges, and lessons from the devastating 2023 Turkey earthquakes

Mert, I.S., Koksal, K.
International Nursing Review, 2025, 72(2), e13023

Article • *Open access*
The Experiences of Nurses as First Responders to Disaster: A Qualitative Study
Açık, C., Yeşilyurt Sevim, T.
Nursing and Health Sciences, 2025, 27(2), e70084

Article • *Open access*
Challenges and Strategies in Maintaining Continuity of Care for Chronic Disease Patients by Emergency Nurses During Disasters
Alanazi, M.A., Shaban, M.
International Nursing Review, 2025, 72(2), e70029

Article
Lived experience of student responders with leadership in a mass casualty simulation
Anderson, M., Reed, C., Watson, A., ... Schmutz, P., Rasmussen, R.
Journal of Professional Nursing, 2025, 58, pp. 83–92

Article
Select Demographic Data, Disaster-Related Experience, and Disaster Attitudes as Predictors of Disaster Preparedness Among Student Nurses: A Descriptive Correlational Study
Tuquero, B.N.G., Tan, R.N.G., Tango, G.J.G., ... Trinidad, C.E.V., Pan, W.K.M.
Public Health Nursing, 2025, 42(3), pp. 1335–1342

Review • *Open access*
Factors Influencing Patient Presentation and Transfer to Hospital Rates during Mass-Gathering Stadium Events: A Scoping Review
Sultana, N., Crilly, J., Ware, R.S., Ranse, J.
Prehospital and Disaster Medicine, 2025, 40(2), pp. 101–113

Article • *Open access*
Being a nurse during an earthquake that affected ten provinces: A qualitative study on experiences and expectations
Şermet Kaya, Ş., Gülnur Erdoğan, E.
International Nursing Review, 2025, 72(1), e13051

Article
Evaluating nurses' psychological and operational preparedness for mass-casualty events in Saudi Arabia
Shubayr, N.
International Nursing Review, 2025, 72(1), e70002

Article • *Open access*
Challenges and needs in disaster preparedness: A qualitative study | Desafíos y necesidades en la preparación ante desastres: un estudio cualitativo
Purnomo, E., Hamid, A.Y.S., Gayatri, D., Setiawan, A.
Salud Ciencia Y Tecnologia, 2025, 5, 1225

Note • *Open access*
Pediatric Emergency Nursing in Gaza: Challenges, Adaptations, and Lessons from a Conflict Zone
Smadi, Z., Ghali, A., Rizek, J., ... Koujah, D., Muhammad, A.
Sage Open Nursing, 2025, 11, 23779608251349070

Article • Article in Press
Being Both an Earthquake Survivor and a Nurse: Türkiye's Twin Earthquakes
Alan, H., Özen Bekar, E., Kavaslar, İ.
Workplace Health and Safety, 2025, 21650799251367357

Article • *Open access*

The double burden of stressful life events among professional nurses: public mass shootings during the COVID-19 pandemic
Imkome, E.-U.
International Journal of Qualitative Studies on Health and Well Being, 2025, 20(1), 2504477

Article
Pre-hospital emergency care personnel's challenges in providing care in mass casualty incidents: A qualitative study
Bijani, M., Javad Moradian, M., Najafi, H., Arbon, P., Abedi, S.
International Emergency Nursing, 2024, 77, 101522

Review • *Open access*
Overcoming challenges in nursing disaster preparedness and response: an umbrella review
Al Thobaity, A.
BMC Nursing, 2024, 23(1), 562

Article
Caring for Women in an Active War Zone
Muhammad, A., Rizek, J.
Journal of Emergency Nursing, 2024, 50(6), pp. 722–726

Article
Investigating perceived core disaster competencies of nurses in Iran: A case study of northwest hospitals
Ziapour, A., Darabi, F., Rostami, F., ... Yildirim, M., Kianipour, N.
Public Health Nursing, 2024, 41(5), pp. 970–978

Review • *Open access*
Common Challenges in the Prehospital Management of Mass-Casualty Incidents: A Systematic Integrative Review
Hugelius, K., Becker, J.
Prehospital and Disaster Medicine, 2024, 39(4), pp. 301–309

Article
Experiences of adolescents affected by earthquakes: A qualitative study
Başkale, H., Solmaz, P.
Journal for Specialists in Pediatric Nursing, 2024, 29(3), e12434

Article
Experiences and psychosocial challenges of volunteer nurses in Turkey devastating earthquake zones: Lessons to be learnt for prevention of health system problems in disasters: A qualitative study
Yanik, D., Ediz, Ç.
Public Health Nursing, 2024, 41(3), pp. 503–513

Review • *Open access*
Using High-Fidelity Virtual Reality for Mass-Casualty Incident Training by First Responders - A Systematic Review of the Literature
Heldring, S., Jirwe, M., Wihlborg, J., Berg, L., Lindström, V.
Prehospital and Disaster Medicine, 2024, 39(1), pp. 94–105

Article • *Open access*
Are you ready? The preparation of Swedish nursing students for disaster nursing in armed conflicts
Lundberg, K., Andersson, U., Andersson, H., Sterner, A.
Nordic Journal of Nursing Research, 2024, 44

Review • *Open access*
A Scoping Review of Nurses' Knowledge and Preparedness in Disaster Management in Saudi Arabia | Una revisión sobre conocimiento y la preparación de las enfermeras en la gestión de desastres en Arabia Saudita
Rashdan Alsolmi, B.
Salud Ciencia Y Tecnologia, 2024, 4, 1003

Article • *Open access*
Development of disaster nursing in Nepal: Opportunities and challenges for advanced practice
Paudel, S., Kanbara, S.
International Nursing Review, 2023, 70(4), pp. 464–472

Article • *Open access*
Management of Mass-Casualty Incidents in Nepal: A Qualitative Case Study of Three District Hospitals in Nepal
Singh, P., Lamine, H., Sapkota, S., Bahattab, A., Eriksson, A.
Prehospital and Disaster Medicine, 2023, 38(5), pp. 606–611

Article
Frequency of Mass Casualty Incidents (MCIs) Responded to by Helicopter Emergency Medical Services (HEMS)
Shekhar, A.C., McCartin, M., Blumen, I.J.
Air Medical Journal, 2023, 42(5), pp. 384–386

Article
Disaster Training for Nurses in Indonesia: Balancing Physical, Psychological, and Managerial Competencies
Susanti, H., Hamid, A.Y.S., Putri, A.F., ... Frida, A., Fadilah, R.
Journal of Continuing Education in Nursing, 2023, 54(8), pp. 378–384

Review
Nursing in the Anthropocene–translating disaster nursing experience into climate crisis nurse education
Richards, C., Holmes, M., Nash, R., Ward, A.

Article • *Open access*
Rethinking the Current Stage-And-Wait Paradigm
Ragoler, M., Radomislensky, I., Dolev, E., Renert, L., Peleg, K.
Prehospital and Disaster Medicine, 2023, 38(2), pp. 185–192

Article • *Open access*
Learning from critical care nurses' wartime experiences and their long-term impacts
Segev, R.
Nursing in Critical Care, 2023, 28(2), pp. 253–260

Article • *Open access*
Collaborative Interprofessional Health Science Student Led Realistic Mass Casualty Incident Simulation
McCrea, D.L., Coghlan, R.C., Champagne-Langabeer, T., Cron, S.
Healthcare Switzerland, 2023, 11(1), 40

Article
Navy En-Route Care in Future Distributed Maritime Operations: A Review of Clinician Capabilities and Roles of Care
Eisenhauer, I.F., Walrath, B.D., Bebarta, V.S., ... Baker, J.B., Schauer, S.G.
Prehospital Emergency Care, 2023, 27(4), pp. 465–472

Article
A Qualitative Study of Disaster Resilience of Chinese Adolescents Five Years after Super Typhoon Rammasun
Yan, Y., Turale, S., Klunklin, P., Klunklin, A.
Pacific Rim International Journal of Nursing Research, 2023, 27(2), pp. 213–229

Article
Attacks on Educational Institutions
Tin, D., Issa, F., Ciottone, G.R.
Prehospital and Disaster Medicine, 2022, 37(3), pp. 333–337

Article • *Open access*
Management of COVID-19 mass casualty incidents in nursing and retirement homes | Managementstrategie für den Massenanfall von Erkrankten/Infizierten in Alten- und Pflegeheimen im Kontext der COVID-19-Pandemie
Schreiber, W., Wolf, P., Bigalke, N., ... Graf, B.M., Dittmar, M.S.
Medizinische Klinik Intensivmedizin Und Notfallmedizin, 2022, 117(4), pp. 289–296

Article • *Open access*
The history of disaster nursing: from Nightingale to nursing in the 21st century
Fletcher, K.A., Reddin, K., Tait, D.
Journal of Research in Nursing, 2022, 27(3), pp. 257–272

Article
Logistic Red Flags in Mass-Casualty Incidents and Disasters: A Problem-Based Approach
Gamberini, L., Imbriaco, G., Ingrassia, P.L., ... Gordini, G., Della Corte, F.
Prehospital and Disaster Medicine, 2022, 37(2), pp. 197–204

Article • *Open access*
Disaster preparedness and core competencies among emergency nurses: A cross-sectional study
Chegini, Z., Arab-Zozani, M., Kakemam, E., ... Nobakht, A., Aziz Karkan, H.
Nursing Open, 2022, 9(2), pp. 1294–1302

Article • *Open access*
Medical coordination rescue members' and ambulance nurses' perspectives on a new model for mass casualty and disaster management and a novel terror attack mitigation approach in the netherlands: A qualitative study
Berben, S.A.A., Vloet, L.C.M., Lischer, F., Pieters, M., De Cock, J.
Prehospital and Disaster Medicine, 2021, 36(5), pp. 519–525

Article
Half-a-century of terrorist attacks: Weapons selection, casualty outcomes, and implications for counter-terrorism medicine
Tin, D., Margus, C., Ciottone, G.R.
Prehospital and Disaster Medicine, 2021, 36(5), pp. 526–530

Article • *Open access*
A qualitative study on researchers' experiences after publishing scientific reports on major incidents, mass-casualty incidents, and disasters
Svensoy, J.N., Nilsson, H., Rimstad, R.
Prehospital and Disaster Medicine, 2021, 36(5), pp. 536–542

Article
The retrospect and prospect of disaster nursing development in China | 我国灾害护理发展的回顾与展望
Saiwen, L., Min, Y., Wentao, L.
Chinese Journal of Practical Nursing, 2021, 37(27), pp. 2084–2087

Article
Volunteer nurses' learning experiences in Ludian County, Yunnan, China: Implication for public health nursing education in a disaster
Li, S., Chen, S., Chan, S.
Public Health Nursing, 2021, 38(3), pp. 419–426

Article
Development of disaster nursing education and training programs in the past 20 years (2000–2019): A systematic review
Loke, A.Y., Guo, C., Molassiotis, A.
Nurse Education Today, 2021, 99, 104809

Article • *Open access*
Nurses' stories from Beirut: The 2020 explosive disaster on top of a pandemic and economic crises
Jabbour, R., Harakeh, M., Dakessian Sailan, S., ... Puzantian, H., Darwish, H.
International Nursing Review, 2021, 68(1), pp. 1–8

Article • *Open access*
Structural Characteristics of Nursing Homes and Social Service Directors that Influence Their Engagement in Disaster Preparedness Processes
Kusmaul, N., Beltran, S., Buckley, T., Gibson, A., Bern-Klug, M.
Journal of Gerontological Social Work, 2021, 64(7), pp. 775–790

Article • Article in Press
Building capacity in times of crisis: Increasing cultural competence of healthcare professionals in the context of the COVID-19
Slobodin, O., Kula, Y., Clempert, N., Cohen, O.
Journal of Clinical Nursing, 2021

Article • *Open access*
An analysis of movement patterns in mass casualty incident simulations
Tolg, B., Lorenz, J.
Advances in Simulation, 2020, 5(1), 27

Article
Reviewing and Reflecting on Nursing During the COVID-19 Pandemic
Chen, S.-L.
Hu Li Za Zhi the Journal of Nursing, 2020, 67(6), pp. 4–5

Article
The Development of PRIMA-A Belgian Prediction Model for Patient Encounters at Mass Gatherings
Spaepen, K., Haenen, W.A.P., Hubloue, I.
Prehospital and Disaster Medicine, 2020, 35(5), pp. 554–560

Article
Telenursing in Incidents and Disasters: A Systematic Review of the Literature
Nejadshafiee, M., Bahaadinbeigy, K., Kazemi, M., Nekoei-Moghadam, M.
Journal of Emergency Nursing, 2020, 46(5), pp. 611–622

Article • *Open access*
Moral Distress among Disaster Responders: What is it?
Gustavsson, M.E., Arnberg, F.K., Juth, N., Von Schreeb, J.
Prehospital and Disaster Medicine, 2020, 35(2), pp. 212–219

Article
Study of Medical Demand-Supply Balance for the Nankai Trough Earthquake
Takada, Y., Otomo, Y.
Prehospital and Disaster Medicine, 2020, 35(2), pp. 160–164

Article
Accuracy of National Early Warning Score 2 (NEWS2) in Prehospital Triage on In-Hospital Early Mortality: A Multi-Center Observational Prospective Cohort Study
Martín-Rodríguez, F., López-Izquierdo, R., Del Pozo Vegas, C., ... Martín, V.M., Villamor, M.A.C.
Prehospital and Disaster Medicine, 2019, 34(6), pp. 610–618

Article
Organizing Health Care Services for the 2017 Athens Marathon, the Authentic: Perspectives on Collaboration among Health and Safety Personnel in the Marathon Command Center
Bistaraki, A., Georgiadis, K., Pyrros, D.G.
Prehospital and Disaster Medicine, 2019, 34(5), pp. 467–472

Article
Triage in Complex, Coordinated Terrorist Attacks
Pepper, M., Archer, F., Moloney, J.
Prehospital and Disaster Medicine, 2019, 34(4), pp. 442–448

Article
Nurses' disaster preparedness and core competencies in Turkey: a descriptive correlational design
Taskiran, G., Baykal, U.
International Nursing Review, 2019, 66(2), pp. 165–175

Review

The challenges and opportunities in disaster nursing education in Turkey
Kalanlar, B.
Journal of Trauma Nursing, 2019, 26(3), pp. 164–170

Article
Human stampedes: An updated review of current literature
De Almeida, M.M., Von Schreeb, J.
Prehospital and Disaster Medicine, 2019, 34(1), pp. 89–94

Article
Challenges of Burn Mass Casualty Incidents in the Prehospital Setting: Lessons From the Formosa Fun Coast Park Color Party
Lin, C.-H., Lin, C.-H., Tai, C.-Y., Lin, Y.-Y., Shih, F.F.-Y.
Prehospital Emergency Care, 2019, 23(1), pp. 44–48

Article • *Open access*
Is there a triage sieve knowledge and application gap between clinical team leaders and their team members?
Cuttance, G., Dansie, K., Rayner, T.
Australasian Journal of Paramedicine, 2019, 16

Article • *Open access*
A needs assessment for simulation-based training of emergency medical providers in Nebraska, USA
Wehbi, N.K., Wani, R., Yang, Y., ... Adams, J., Paulman, P.
Advances in Simulation, 2018, 3(1), 22

Review • *Open access*
The role of hospital medicine in emergency preparedness: A framework for hospitalist leadership in disaster preparedness, response, and recovery
Persoff, J., Ornoff, D., Little, C.
Journal of Hospital Medicine, 2018, 13(10), pp. 713–718

Article
Flood disaster preparedness experiences of hospital personnel in Thailand: A qualitative study
Rattanakanlaya, K., Sukonthasarn, A., Wangsrikhun, S., Chanprasit, C.
Australasian Emergency Care, 2018, 21(3), pp. 87–92

Article
Why a disaster is not just normal business ramped up: Disaster response among ED nurses
Hammad, K.S., Arbon, P., Gebbie, K., Hutton, A.
Australasian Emergency Care, 2018, 21(1), pp. 36–41

Book Chapter
Hospital Planning and Response to Sudden Mass Casualty Incidents
Lynn, M.
Disasters and Mass Casualty Incidents the Nuts and Bolts of Preparedness and Response to Protracted and Sudden Onset Emergencies, 2018, pp. 29–58

Book
Disasters and Mass Casualty Incidents: The Nuts and Bolts of Preparedness and Response to Protracted and Sudden Onset Emergencies, Second Edition
Lynn, M.
Disasters and Mass Casualty Incidents the Nuts and Bolts of Preparedness and Response to Protracted and Sudden Onset Emergencies Second Edition, 2018, pp. 1–171

Book Chapter
Planning Exercises and Drills for Sudden Mass Casualty Incidents
Lynn, M.
Disasters and Mass Casualty Incidents the Nuts and Bolts of Preparedness and Response to Protracted and Sudden Onset Emergencies, 2018, pp. 111–114

Book Chapter
General Information
Lynn, M.
Disasters and Mass Casualty Incidents the Nuts and Bolts of Preparedness and Response to Protracted and Sudden Onset Emergencies, 2018, pp. 3–9

Book Chapter
Challenges with Deceased and Body Parts Identification
Lynn, M.
Disasters and Mass Casualty Incidents the Nuts and Bolts of Preparedness and Response to Protracted and Sudden Onset Emergencies, 2018, pp. 109–110

Article
Could the internet of things be used to enhance student nurses' experiences in a disaster simulation?
Laplante, N.L., Laplante, P.A., Voas, J.M.
Online Journal of Nursing Informatics, 2018, 22(1), 2

Article • *Open access*
Assessing and Improving Hospital Mass-Casualty Preparedness: A No-Notice Exercise
Waxman, D.A., Chan, E.W., Pillemer, F., ... Abir, M., Nelson, C.
Prehospital and Disaster Medicine, 2017, 32(6), pp. 662–666

Article
Disaster nursing experiences of Chinese nurses responding to the Sichuan Ya'an earthquake
Li, Y.H., Li, S.J., Chen, S.H., ... Jin, Z.H., Zheng, X.Y.
International Nursing Review, 2017, 64(2), pp. 309–317

Article
Estimation of the demand for hospital care after a possible high-magnitude earthquake in the City of Lima, Peru
Bambarén, C., Uyen, A., Rodriguez, M.
Prehospital and Disaster Medicine, 2017, 32(1), pp. 106–111

Article
Education for developing and sustaining a health care workforce for disaster readiness
Langan, J.C., Lavin, R., Wolgast, K.A., Veenema, T.G.
Nursing Administration Quarterly, 2017, 41(2), pp. 118–127

Article
Use of Medical Reserve Corps Volunteers in a Hospital-based Disaster Exercise
Gist, R., Daniel, P., Grock, A., ... Roblin, P., Arquilla, B.
Prehospital and Disaster Medicine, 2016, 31(3), pp. 259–262

Article
A case study of the high-speed train crash outside Santiago de Compostela, Galicia, Spain
Forsberg, R., V'zquez, J.A.I.
Prehospital and Disaster Medicine, 2016, 31(2), pp. 163–168

Article
Nurses as Leaders in Disaster Preparedness and Response-A Call to Action
Veenema, T.G., Griffin, A., Gable, A.R., ... Dobalian, A., Larson, E.
Journal of Nursing Scholarship, 2016, 48(2), pp. 187–200

Article
Integration of leadership competencies in a community health simulation
Martin, D.R., Furr, S.B., Lane, S.H., Bramlett, M.
British Journal of Nursing, 2016, 25(14), pp. 792–794

Article
Experiencing emergency medical services at Hajj
Leggio, W.J., Mobrad, A., D'Alessandro, K.J., ... Sami, M.A., Raynovich, W.

Australasian Journal of Paramedicine, 2016, 13(4)

Article
Cross-sectional survey of the disaster preparedness of nurses across the Asia-Pacific region
Usher, K., Mills, J., West, C., ... Buettner, P., Woods, C.
Nursing and Health Sciences, 2015, 17(4), pp. 434–443

Article
Mass-gathering Events: The Public Health Challenge of the Kumbh Mela 2013
Dwivedi, S., Cariappa, M.P.
Prehospital and Disaster Medicine, 2015, 30(6), pp. 621–624

Article
Disaster nursing skills, knowledge and attitudes required in earthquake relief: Implications for nursing education
Yan, Y.E., Turale, S., Stone, T., Petrini, M.
International Nursing Review, 2015, 62(3), pp. 351–359
Article
First responder accuracy using SALT after brief initial training
Lee, C.W.C., Mcleod, S.L., Peddle, M.B.
Prehospital and Disaster Medicine, 2015, 30(5), pp. 447–451
Review
Ethical and legal challenges associated with disaster nursing
Aliakbari, F., Hammad, K., Bahrami, M., Aein, F.
Nursing Ethics, 2015, 22(4), pp. 493–503

Article
Barriers to pediatric disaster triage: A qualitative investigation
Koziel, J.R., Meckler, G., Brown, L., ... Walsh, B., Cicero, M.X.
Prehospital Emergency Care, 2015, 19(2), pp. 279–286

Article
A better START for low-acuity victims: Data-driven refinement of mass casualty triage
Cross, K.P., Petry, M.J., Cicero, M.X.
Prehospital Emergency Care, 2015, 19(2), pp. 272–278

Article
Recent Advances in Medical Device Triage Technologies for Chemical, Biological, Radiological, and Nuclear Events
Lansdowne, K., Scully, C.G., Galeotti, L., ... Marcozzi, D., Strauss, D.G.
Prehospital and Disaster Medicine, 2015, 30(3), pp. 320–323

Article
Building health care system capacity: Training health care professionals in disaster preparedness health care coalitions
Walsh, L., Craddock, H., Gulley, K., Strauss-Riggs, K., Schor, K.W.
Prehospital and Disaster Medicine, 2015, 30(2), pp. 123–130

Article
Care concepts in mass casualty incidents and disasters: Concept for primary care clinic | Versorgungskonzepte im Großschadens- und Katastrophenfall: Das Konzept der Erstversorgungsklinik
Adams, H.A., Flemming, A., Lange, C., Koppert, W., Krettek, C.
Medizinische Klinik Intensivmedizin Und Notfallmedizin, 2015, 110(1), pp. 27–36

Article
A large-scale accident in Alpine terrain | Großunfall im alpinen Gelände
Wildner, M., Paal, P.
Medizinische Klinik Intensivmedizin Und Notfallmedizin, 2015, 110(1), pp. 21–26

Article

Chinese nurses' relief experiences following two earthquakes: Implications for disaster education and policy development
Wenji, Z., Turale, S., Stone, T.E., Petrini, M.A.
Nurse Education in Practice, 2015, 15(1), pp. 75–81
69
Review
Disaster nursing in Iran: Challenges and opportunities
Zarea, K., Beiranvand, S., Sheini-Jaberi, P., Nikbakht-Nasrabadi, A.
Australasian Emergency Nursing Journal, 2014, 17(4), pp. 190–196

Article
Mexico city's petroleos mexicanos explosion: Disaster management and air medical transport
Urquieta, E., Varon, J.
Air Medical Journal, 2014, 33(6), pp. 309–313

Article
Disaster preparedness and response: Challenges for Australian public health nurses - A literature review
Rokkas, P., Cornell, V., Steenkamp, M.
Nursing and Health Sciences, 2014, 16(1), pp. 60–66

Article
Nurses' experiences of ethical preparedness for public health emergencies and healthcare disasters: A systematic review of qualitative evidence
Johnstone, M.-J., Turale, S.
Nursing and Health Sciences, 2014, 16(1), pp. 67–77

Article
Acute incident rapid response at a mass-gathering event through comprehensive planning systems: A case report from the 2013 shamrock shuffle
Başdere, M., Ross, C., Chan, J.L., ... Smilowitz, K., Chiampas, G.
Prehospital and Disaster Medicine, 2014, 29(3), pp. 320–325

Article
Medical support at a large-scale motorsports mass-gathering event: The inaugural formula one united states grand prix in Austin, Texas
Sabra, J.P., Cabañas, J.G., Bedolla, J., ... Ziebell, C., Olvey, S.
Prehospital and Disaster Medicine, 2014, 29(4), pp. 392–398

Article
Managing multiple-casualty incidents: a rural medical preparedness training assessment.
Glow, S.D., Colucci, V.J., Allington, D.R., Noonan, C.W., Hall, E.C.
Prehospital and Disaster Medicine, 2013, 28(4), pp. 334–341

Article
Online Victim Tracking and Tracing System (ViTTS) for major incident casualties.
Marres, G.M., Taal, L., Bemelman, M., Bouman, J., Leenen, L.P.
Prehospital and Disaster Medicine, 2013, 28(5), pp. 445–453

Article
Hospital disaster preparedness as measured by functional capacity: a comparison between Iran and Sweden.
Djalali, A., Castren, M., Khankeh, H., ... Ohlen, G., Kurland, L.
Prehospital and Disaster Medicine, 2013, 28(5), pp. 454–461

Article
Nursing children after a disaster: A qualitative study of nurse volunteers and children after the Haiti earthquake
Sloand, E., Ho, G., Klimmek, R., Pho, A., Kub, J.
Journal for Specialists in Pediatric Nursing, 2012, 17(3), pp. 242–253

Article

Increased situation awareness in major incidents'radio frequency identification (RFID) Technique: A promising tool
Jokela, J., Rådestad, M., Gryth, D., ... Luoto, M., Castrén, M.
Prehospital and Disaster Medicine, 2012, 27(1), pp. 81–87

Article
A survey of the practice of nurses' skills in Wenchuan earthquake disaster sites: Implications for disaster training
Yin, H., He, H., Arbon, P., Zhu, J.
Journal of Advanced Nursing, 2011, 67(10), pp. 2231–2238

Article
Research issues for nursing and midwifery contributions in emergencies
Weiner, E.
Prehospital and Disaster Medicine, 2011, 26(2), pp. 109–113

Article
The Haiti earthquake: the provision of wound care for mass casualties utilizing negative-pressure wound therapy.
Gabriel, A., Gialich, S., Kirk, J., ... Gabriel, C., Gupta, S.
Advances in Skin Wound Care, 2011, 24(10), pp. 456–462

Article
Development and evaluation of an undergraduate training course for developing international council of nurses disaster nursing competencies in China
Chan, S.S.S., Chan, W.-S., Cheng, Y., ... Yip, A.L.K., Pang, S.M.C.
Journal of Nursing Scholarship, 2010, 42(4), pp. 405–413

Article
Taking seriously the what then? question: an ethical framework for the responsible management of medical disasters.
McCullough, L.B.
Journal of Clinical Ethics, 2010, 21(4), pp. 321–327

Article
Disaster response: Essential competence for nurses
Lee, C.-L., Chiang, L.-C.
Journal of Nursing, 2010, 57(3), pp. 5–10

Article
Chinese nurses' experience in the Wenchuan earthquake relief
Yang, Y.-N., Xiao, L.D., Cheng, H.-Y., Zhu, J.-C., Arbon, P.
International Nursing Review, 2010, 57(2), pp. 217–223

Book Chapter
Disaster nursing educational competencies
Slepski, L.A., Littleton-Kearney, M.T.
International Disaster Nursing, 2010, pp. 549–560

Book Chapter
Explosive event Preparedness/Response
Agostini, A.
International Disaster Nursing, 2010, pp. 253–264

Book Chapter
International disaster response
Briggs, S.M.
International Disaster Nursing, 2010, pp. 351–364

Book Chapter
Disaster nursing research
Whitehead, D., Arbon, P.

International Disaster Nursing, 2010, pp. 561–582

Article
Botulism Questionnaire: A tactical tool for community use in a mass casualty incident
Burkholder-Allen, K., Rega, P., Bork, C., Budd, C.
Nursing and Health Sciences, 2009, 11(4), pp. 374–377

Article
Disaster education and training of emergency nurses in South Australia
Duong, K.
Australasian Emergency Nursing Journal, 2009, 12(3), pp. 86–92

Article
Ethical considerations of research in disaster-stricken populations
Jesus, J.E., Michael, G.E.
Prehospital and Disaster Medicine, 2009, 24(2), pp. 109–114

Article
Mass-casualty triage training for international healthcare workers in the Asia-pacific region using manikin-based simulations
Vincent, D.S., Berg, B.W., Ikegami, K.
Prehospital and Disaster Medicine, 2009, 24(3), pp. 206–213

Review
Personal protection during resuscitation of casualties contaminated with chemical or biological warfare agents-A survey of medical first receivers
Brinker, A., Prior, K., Schumacher, J.
Prehospital and Disaster Medicine, 2009, 24(6), pp. 525–528

Review
Maternal and Newborn Care During Disasters: Thinking Outside the Hospital Paradigm
Pfeiffer, J., Avery, M.D., Benbenek, M., ... Wachdorf, C.M., O'Boyle, C.
Nursing Clinics of North America, 2008, 43(3), pp. 449–467

Article
Lessons public health professionals learned from past disasters
Rebmann, T., Carrico, R., English, J.F.
Public Health Nursing, 2008, 25(4), pp. 344–352

Article
Challenges and promises for nurse education curriculum development in Kosovo: Results of an accidental ethnography
Goepp, J.G., Johnson, T.D., Maddow, C.L.
Nurse Education Today, 2008, 28(4), pp. 419–426

Article
Use of shuttered hospitals to expand surge capacity
Zane, R.D., Biddinger, P., Ide, L., ... Carr, D., Hassol, A.
Prehospital and Disaster Medicine, 2008, 23(2), pp. 121–127

Review
Decontamination of multiple casualties who are chemically contaminated: A challenge for acute hospitals
Clarke, S.F.J., Chilcott, R.P., Wilson, J.C., ... Baker, D.J., Hallett, A.
Prehospital and Disaster Medicine, 2008, 23(2), pp. 175–181

Review
Mass-casualty triage: Time for an evidence-based approach
Jenkins, J.L., McCarthy, M.L., Sauer, L.M., ... Thomas, T.L., Hsu, E.B.
Prehospital and Disaster Medicine, 2008, 23(1), pp. 3–8

Article
Lessons learned from chlorine intoxications in swimming pools: The challenge of pediatric mass toxicological events
Lehavi, O., Leiba, A., Dahan, Y., ... Levi, Y., Bar-Dayan, Y.
Prehospital and Disaster Medicine, 2008, 23(1), pp. 90–95

Article • *Open access*
Mid-term report on the project disaster nursing in a ubiquitous society in the academic years 2003 and 2004
Yamamoto, A.
Japan Journal of Nursing Science, 2006, 3(1), pp. 65–69

Article
A four-step approach for establishment of a national medical response to mega-terrorism
Leiba, A., Blumenfeld, A., Hourvitz, A., ... Levi, Y., Bar-Dayan, Y.
Prehospital and Disaster Medicine, 2006, 21(6), pp. 436–440

Article
Nursing students' perceptions about disaster nursing
Jennings-Sanders, A., Frisch, N., Wing, S.
Disaster Management and Response, 2005, 3(3), pp. 80–85

Article
Challenges of major incident management when excess resources are allocated: Experiences from a mass casualty incident after roof collapse of a military command center
Romundstad, L., Sundnes, K.O., Pillgram-Larsen, J., Røste, G.K., Gilbert, M.
Prehospital and Disaster Medicine, 2004, 19(2), pp. 179–184

WoS

N= 75, included 0, duplicates 23, removed 52
Triage ethics in mass casualty incident simulation: A phenomenological exploration
Watson, AL; Drake, J; (...); Rasmussen, R
Jun 2025
NURSING ETHICS
32 (4) , pp.1313-1326
Enriched Cited References
Background Disaster scenarios challenge both novice and experienced nurses to navigate complex ethical dilemmas in resource-limited environments. Traditional nursing education often leaves new nurses unprepared for the ethical demands of disaster nursing. Utilitarianism must often guide triage ethics and decision-making. There is a critical need to equip nursing students with these ethical comp
Show more

2
Unveiling the heart of disaster nursing: A qualitative study on motivations, challenges, and lessons from the devastating 2023 Turkey earthquakes
Mert, IS and Koksal, K
Jun 2025
INTERNATIONAL NURSING REVIEW
72 (2)
Enriched Cited References
BackgroundTurkey has faced a notable escalation in earthquake disasters in the last two decades. Despite initiating a health and disaster management system, nurses' pivotal roles and experiences in handling such crises have been disregarded.AimThis qualitative study analyzed nurses' experiences before, during, and after deployment in response to the 2023 Turkey earthquakes to enhance disaster-r
Show more

3
Development of disaster nursing in Nepal: Opportunities and challenges for advanced practice
Paudel, S and Kanbara, S
Dec 2023
INTERNATIONAL NURSING REVIEW
70 (4) , pp.464-472
Enriched Cited References
AimThis article discusses the evolution of the field of disaster nursing in Nepal and its challenges and ways to protect the health and well-being of the community during disasters. It also discusses the alignment of field activities with the International Council of Nurses' (ICN) competencies in disaster nursing.BackgroundDisaster nursing has become increasingly important due to the increasing

---

4
Nursing in the Anthropocene-translating disaster nursing experience into climate crisis nurse education
Semantic search result
Richards, C; Holmes, M; (...); Ward, A
Jul 2023
TEACHING AND LEARNING IN NURSING
18 (3) , pp.e113-e121
Healthcare systems in many Organization for Economic Cooperation and Development Countries (OECD) are ill prepared for minimizing the risks and withstanding the impacts of natural disasters caused by climate change. In the 21st century, all nursing specialties will be affected by escalating natural disasters as practice is adapted for the Anthropocene. This rapid review defines the current evid
Show more

5
DISASTER NURSING FOR EARLY CAREER EMERGENCY NURSES
Semantic search result
Rizek, J
Jul 2025

JOURNAL OF EMERGENCY NURSING
51 (4) , pp.537-547
Disaster nursing has emerged as a critical subspecialty within the nursing profession, addressing the rising frequency and severity of natural, environmental, and public health disasters. Recognizing its significance, disaster nursing competencies present an opportunity for further standardization within academic nursing programs. Key aspects include community health promotion, critical decisio
Show more

6
Disaster Nursing Competencies in a Time of Global Conflicts and Climate Crises: A Cross-Sectional Survey Study
Semantic search result
Licen, S and Prosen, M
Jun 2025
INTERNATIONAL NURSING REVIEW
72 (2)
Enriched Cited References
AimThe aim of this study was to explore the disaster-related nursing competencies among Slovenian nurses, focusing on core competencies, barriers to competency development and roles in disaster management. It also examined the predictors of these competencies, including participation in training, emergency drills, qualifications and leadership roles.BackgroundGlobal conflicts and climate crises

---

7
The Challenges and Opportunities in Disaster Nursing Education in Turkey
Kalanlar, B
May-jun 2019
JOURNAL OF TRAUMA NURSING
26 (3) , pp.164-170
Despite the key role of nurses in the disaster management process, there are still few studies in Turkey that have investigated the unique role of nurses in disasters. The aim of this study is to assess the challenges and opportunities in disaster nursing education in Turkey. In this context, this article will discuss disaster nursing, the role of nurses in disasters, and topics related to disa
Show more

8
Ethical and legal challenges associated with disaster nursing
Aliakbari, F; Hammad, K; (...); Aein, F
Jun 2015
NURSING ETHICS
22 (4) , pp.493-503
Background: In disaster situations, nurses may face new and unfamiliar ethical and legal challenges not common in their everyday practice.
Research question/objectives/hypothesis: The aim of this study was to explore Iranian nurses' experience of disaster response and their perception of the competencies required by nurses in this environment.
Research design: This article discusses

9
Disaster nursing experiences of Chinese nurses responding to the Sichuan Ya'an earthquake
Li, YH; Li, SJ; (...); Zheng, XY
Jun 2017
INTERNATIONAL NURSING REVIEW
64 (2) , pp.309-317
Aim: The aim of this study was to investigate the disaster experiences of nurses called to assist survivors one month after the 2013 Ya'an earthquake.
Background: China has experienced an increasing number of earthquake disasters in the past four decades. Although a health and disaster management system was initiated after the 2008 Wenchuan earthquake, nurses' roles and experiences in a d
Show more

10

Overcoming challenges in nursing disaster preparedness and response: an umbrella review
Semantic search result
Al Thobaity, A
Aug 14 2024
BMC NURSING
23 (1)
Disaster nursing plays a vital role in addressing the health needs of vulnerable populations affected by large scale emergencies. However, disaster nursing faces numerous challenges, including preparedness, logistics, education, ethics, recovery and legalities. To enhance healthcare system effectiveness during crises, it is essential to overcome these issues. This umbrella review, conducted usi
Show more

11
Chinese nurses' relief experiences following two earthquakes: Implications for disaster education and policy development
Zhou, WJ; Turale, S; (...); Petrini, MA
Jan 2015
NURSE EDUCATION IN PRACTICE
15 (1) , pp.75-81
Disasters require well trained nurses but disaster nursing education is very limited in China and evidence is urgently required for future planning and implementation of specialized disaster education. This describes the themes arising from narratives of Chinese registered nurses who worked in disaster relief after two major earthquakes. In-depth interviews were held with 12 registered nurses f
Show more

12
Teaching Disaster Nursing Competencies: Strategies to Succeed
Semantic search result
Langan, JC and Moore, KS
Jun 2025
JOURNAL OF NURSING EDUCATION
64 (6)
Background: Disaster education for nurses at all levels is imperative to improve the ability for communities to be prepared to respond and recover from disasters and public health emergencies. A variety of educational strategies are available to deliver this vital content. Method: A variety of strategies to deliver necessary disaster nursing education are discussed. The development of online di

---

13
Nurses' competence levels in disaster nursing management in Turkey: A comparative cross-sectional study
Demirtas, H and Altuntas, S
Sep 2024
INTERNATIONAL NURSING REVIEW
71 (3) , pp.556-562
Enriched Cited References
AimThis study aimed to determine nurses' views of their competence in disaster nursing management. BackgroundIt is an important responsibility of nurses to have the necessary knowledge and skills for the management of disasters and to be prepared for disasters. For this responsibility to be effectively demonstrated, it is important to determine the disaster management competencies of the nurses
Show more

14
Status and influencing factors of clinical nurses' disaster nursing competency: a multicenter cross-sectional study
Semantic search result
Zhu, D; Li, YJ; (...); Chen, Y
Apr 5 2025
BMC NURSING
24 (1)
Enriched Cited References
Objectives This study aimed to assess the competency of clinical nurses in disaster nursing in Jiangsu Province, China. Background Clinical nurses represent the largest proportion of the healthcare workforce and often serve

as the primary responders in disaster management. Their competencies in disaster nursing play a pivotal role in ensuring the quality and accuracy of disaster-related care. H
Show more

15
Disaster nursing training needs among different levels of clinical nurses in China: A cross-sectional study with educational implications
Semantic search result
He, JJ; Li, CY; (...); Tao, J
Jul 2025
TEACHING AND LEARNING IN NURSING
20 (3) , pp.e617-e622
Background: Disasters have occurred more and more frequently worldwide, and the role of nurses during disasters has been highlighted to an unprecedented degree. Tailored training programs are called to be provided for nurses at different levels who assume different roles in disasters. While there have been few studies examining the gap between different levels of clinical nurses' existing disas
Show more

16
Development of disaster nursing education and training programs in the past 20 years (2000-2019): A systematic review
Semantic search result
Loke, AY; Guo, CL and Molassiotis, A
Apr 2021
NURSE EDUCATION TODAY
99
Background: Nurses play a pivotal role in disaster management across the globe. With the call for all nurses to be prepared for disasters, disaster nursing education and training programs have expanded globally. However, a clear picture of the development and coverage of disaster nursing education and training programs is lacking. Objectives: This study aimed to establish an overall picture of
Show more

17
The history of disaster nursing: from Nightingale to nursing in the 21st century
Semantic search result
Fletcher, KA; Reddin, K and Tait, D
May 2022
JOURNAL OF RESEARCH IN NURSING
27 (3) , pp.257-272
Enriched Cited References
Background: Nurses have a rich history in performing their duty both domestically and internationally in response to a disaster. Comprising the largest proportion of the healthcare workforce, nurses possess a unique opportunity to inform disaster planning and management. With the ongoing threat from COVID-19 and continuing conflict, humanitarian aid needs, epidemics and natural disasters; the c
Show more
Related records

18
Disaster Nursing Competency of Intensive Care Nurses in Jinan, China: A Multicenter Cross-Sectional Study
Semantic search result
Jiang, M; Sun, M; (...); Li, RJ
Jun 2022
JOURNAL OF NURSING RESEARCH
30 (3)
Enriched Cited References
Background Patients in disaster areas require the most urgent assistance. In recent large-scale natural disasters, intensive care nurses have served as an important reserve component of disaster response teams. In disaster nursing, ability and attitude directly affect the quality and effectiveness of disaster rescues. However, few studies have examined the disaster nursing competency of intensi
Show more

19
An illumination of the ICN's core competencies in disaster nursing version 2.0: Advanced nursing response to COVID-19 outbreak in China
Semantic search result
Mao, XR; Yang, Q; (...); Loke, AY
Apr 2021
JOURNAL OF NURSING MANAGEMENT
29 (3) , pp.412-420
Enriched Cited References
Aim This study aims to report on the actions and incident management of the advanced practice nurses of a disaster operation team who were deployed in response to the COVID-19 outbreak, and to explore how it illustrated the Core Competencies in Disaster Nursing Version 2.0 delineated by the International Council of Nurses in 2019.
Methods This is a descriptive study. The participants (res

20
Correlations between emergency code awareness and disaster nursing competencies among clinical nurses: A cross-sectional study
Semantic search result
Jeong, S and Lee, OG
Sep 2020
JOURNAL OF NURSING MANAGEMENT
28 (6) , pp.1326-1334
Enriched Cited References
Aims This study identified clinical nurses' awareness of emergency codes and disaster nursing competencies and investigated the relationships between these variables. Background Nurses are on the front lines of disaster and emergency response; however, they report not feeling confident about their disaster preparedness. Methods Participants included 234 nurses working at a general hospital in K
Show more

21
Factors influencing disaster nursing core competencies of emergency nurses
Semantic search result
Park, HY and Kim, JS
Oct 2017
APPLIED NURSING RESEARCH
37 , pp.1-5
Background: Emergency nurses are expected to provide required nursing services by using their professional expertise to reduce the risk posed by disasters. Thus, emergency nurses' disaster nursing core competencies are essential for coping with disasters. The purpose of the study reported here was to identify factors influencing the disaster nursing core competencies of emergency nurses.
Show more

22
A new scale for disaster nursing core competencies: Development and psychometric testing
Semantic search result
Al Thobaity, A; Williams, B and Plummer, V
Feb 2016
AUSTRALASIAN EMERGENCY NURSING JOURNAL 19 (1) , pp.11-19
Background: All nurses must have core competencies in preparing for, responding to and recovering from a disaster. In the Kingdom of Saudi Arabia (KSA), as in many other countries, disaster nursing core competencies are not fully understood and lack reliable, validated tools. Thus, it is imperative to develop a scale for exploring disaster nursing core competencies, roles and barriers in the KS
Show more

23
Disaster nursing skills, knowledge and attitudes required in earthquake relief: Implications for nursing education
Yan, YE; Turale, S; (...); Petrini, M
Sep 2015

INTERNATIONAL NURSING REVIEW
62 (3) , pp.351-359
BackgroundGlobally, nurses becoming more aware of getting better prepared for disaster relief, but in China, disaster nursing knowledge, courses and research are still limited.
IntroductionChina has long been prone to disasters, but disaster nursing education and training is in its infancy.
AimThis study explored the skills, knowledge and attitudes required by registered nurses from
Show more

24
An Important Concept of Protecting Public Health in Disaster Situations: Disaster Nursing
Semantic search result
Kalanlar, B and Kubilay, G
2015
FLORENCE NIGHTINGALE JOURNAL OF NURSING-FLORENCE NIGHTINGALE HEMSIRELIK DERGISI 23 (1) , pp.57-65
Since Florence Nightingale, nurses have undertaken a variety of roles in disaster management with their evaluation making skills, prioritization, communication, cooperation and critical thinking skills. Therefore, nurses are held responsible for gaining necessary knowledge and skills in disaster management and to be prepared for possible disasters. Nurses whatever their area of expertise should
Show more

25
All the Resources was Gone The Environmental Context of Disaster Nursing
Semantic search result
Ruskie, SE
Dec 2016
NURSING CLINICS OF NORTH AMERICA
51 (4) , pp.569-+
US nurses are not prepared for the altered conditions of the disaster environment, nor has the context of providing disaster nursing care been a focus of disaster research. Using an existential phenomenologic approach, US nurses described the "not normal" conditions of the disaster environment they experienced as physically and emotionally challenging, because of the reduced infrastructural cap
Show more

26
Disaster management competence, disaster preparedness belief, and disaster preparedness relationship: Nurses after the 2023 Turkey earthquake
Semantic search result
Kaya, SS and Erdogan, EG
Mar 2025
INTERNATIONAL NURSING REVIEW
72 (1)
Enriched Cited References
AimTo examine the relationship between nurses' competency levels in disaster nursing management, their disaster preparedness and disaster preparedness beliefs.BackgroundNurses' competency in disaster nursing management makes disaster response easier. Factors that may affect this reason should be examined.MethodsThe study was conducted between April and July 2023 with nurses working in a provinc
Show more

27
Development and Evaluation of an Immersive Cinematic Escape Room for Disaster Preparedness and Self-Efficacy Among Nurses
Semantic search result
Hsiao, CC; Huang, CY; (...); Cheng, SF
Jun 2024
CLINICAL SIMULATION IN NURSING
91
Background: The occurrence of natural disasters is unpredictable. Nurses need to have the core competency of disaster nursing to effectively respond to natural disasters. This study aimed to develop and evaluate the

effectiveness of an immersive cinematic escape room (ICER) instructional approach in disaster preparedness and self -efficacy in nurses. Method: This quasi -experimental research de
Show more

28
Disaster Education in Nursing Education: Is it Enough? A Literature Review
Semantic search result
Taylor, J
Apr 2025
JOURNAL OF NURSING EDUCATION
64 (4)
Background: Disasters are major events that have significant effects on individuals, communities, and health care systems. Nurses are valuable resources that have a role in disaster response. However, barriers in disaster education in nursing programs negatively affect nurses' knowledge and skills in disaster preparedness and response. Method: A literature review was conducted to better underst
Show more

29
Being Both an Earthquake Survivor and a Nurse: Türkiye's Twin Earthquakes
Alan, H; Bekar, EÖ and Kavaslar, I
Sep 2025 (Early Access)
WORKPLACE HEALTH & SAFETY
Enriched Cited References
Background: In February 2023, twin earthquakes struck 11 provinces in T & uuml;rkiye, devastating infrastructure and healthcare services. Nurses, as both survivors and frontline responders, faced extreme physical, emotional, and organizational challenges. Their experiences offer critical insights into disaster-related occupational risks and the structural gaps in worker health and safety. This
Show more

30
Nurses as Leaders in Disaster Preparedness and ResponseA Call to Action
Veenema, TG; Griffin, A; (...); Larson, E
Mar 2016
JOURNAL OF NURSING SCHOLARSHIP
48 (2) , pp.187-200
PurposeTo develop a vision for the future of disaster nursing, identify barriers and facilitators to achieving the vision, and develop recommendations for nursing practice, education, policy, and research.
Design and MethodsA series of semistructured conference calls were conducted with 14 national subject matter experts to generate relevant concepts regarding national nursing workforce p
Show more

31
Readiness of hospital nurses for disaster responses in Taiwan: A cross-sectional study
Semantic search result
Tzeng, WC; Feng, HP; (...); Lee, CL
Dec 2016
NURSE EDUCATION TODAY
47 , pp.37-42
Background: Because patients in disaster areas require the most critical care, mobilising hospital nurses has become a pivotal strategy. Given the importance of disaster nursing training programmes, understanding how well prepared hospital nurses are to provide disaster care is vital.
Objectives: This paper analyses the perceived readiness of hospital nurses for a disaster response and th
Show more

32
Preparing next generation of nurses to care for disaster affected population: A call to action in nursing curriculum
Semantic search result
Hirani, SAA
Oct 2023

TEACHING AND LEARNING IN NURSING
18 (4) , pp.492-495
Disasters can occur anywhere and without warning, requiring proactive action to reduce errors and save lives. Nurses are often the first responders during times of disasters who play a vital role in preserving life and sustaining all aspects of the health of the disaster-affected community. The inclusion of disaster education in the nursing curriculum is vital to prepare nurses who are competen
Show more

33
Sustainable disaster risk reduction training model for nurses: A descriptive qualitative approach
Semantic search result
Hamid, AYS; Chandra, YA; (...); Yulianingsih, Y
May 2023
NURSE EDUCATION IN PRACTICE
69
Aim: To develop a sustainable disaster risk reduction training model for nurses and identify its key features. Background: Available disaster nursing education and training programmes have focused on improving the competency of nurses in all four disaster phases, including mitigation, preparedness, response and recovery. However, a limited programme is available that integrates nurses' competen
Show more

34
Emergency department registered nurses overestimate their disaster competency: A cross-sectional study
Semantic search result
Murphy, JP; Kurland, L; (...); Rüter, A
Sep 2021
INTERNATIONAL EMERGENCY NURSING
58
Enriched Cited References
Background: Major incidents continue to pose a threat to health care systems by overwhelming them with a sudden surge of patients. A major factor impacting a hospital's surge capacity is the skills, abilities, and knowledge of emergency department (ED) registered nurses (RN). The level of disaster nursing competency they possess affects patient safety and outcome. ED RNs' ability to accurately
Show more

35
Volunteer nurses' learning experiences in Ludian County, Yunnan, China: Implication for public health nursing education in a disaster
Li, SA; Chen, SH and Chan, S
May 2021
PUBLIC HEALTH NURSING
38 (3) , pp.419-426
Enriched Cited References
Objective To describe the learning impacts made by graduate nurse volunteers who worked in a field hospital 1 month following the 2014 Ludian earthquake in Yunnan, China.
Methods A qualitative descriptive approach was adopted. The volunteers were 23 final year part-time students enrolled in the Master of Science in Disaster Nursing program offered by the School of Nursing. Data were colle
Show more

36
Experiences and psychosocial challenges of volunteer nurses in Turkey devastating earthquake zones: Lessons to be learnt for prevention of health system problems in disasters: A qualitative study
Yanik, D and Ediz, C
May 2024
PUBLIC HEALTH NURSING
41 (3) , pp.503-513
Enriched Cited References
ObjectiveThis research aimed to investigate the experiences and psychosocial challenges encountered by volunteer nurses who provided care in the zones affected by the devastating earthquake that struck Turkey in

2023.MethodsThis qualitative study involved semistructured, in-depth interviews with 15 volunteer nurses who were actively working in earthquake-affected regions. The study was conducte
Show more

37
Pediatric Emergency Nursing in Gaza: Challenges, Adaptations, and Lessons from a Conflict Zone
Smadi, Z; Ghali, A; (...); Muhammad, A
2025
SAGE OPEN NURSING
11
Pediatric emergency medicine faces numerous challenges in regions affected by prolonged conflict, such as Gaza. Nurses in Gaza work in overcrowded emergency departments, without access to pediatric-specific supplies or medications, yet they demonstrate remarkable resilience and adaptability, developing innovative solutions to deliver lifesaving care. Therefore, pediatric nurses in conflict sett
Show more

38
Challenges and Strategies in Maintaining Continuity of Care for Chronic Disease Patients by Emergency Nurses During Disasters
Alanazi, MA and Shaban, M
Jun 2025
INTERNATIONAL NURSING REVIEW
72 (2)
Enriched Cited References
AimThis study aimed to explore the challenges emergency nurses face in providing continuity of care for chronic disease patients during disasters in the Northern Region of Saudi Arabia and the strategies they employ to overcome these challenges.BackgroundEmergency nurses play a pivotal role in disaster response, particularly in managing care for patients with chronic diseases. Resource shortage
Show more

39
Strengthening emergency department response to chemical, biological, radiological, and nuclear disasters: A scoping review
Ranse, J; Mackie, B; (...); Hammad, K
Mar 2025
AUSTRALASIAN EMERGENCY CARE
28 (1) , pp.37-47
Introduction: Natural hazards resulting in disasters are increasing globally, impacting communities and disrupting industries. In addition to planning for these natural hazard disasters, emergency departments (EDs) should prepare for chemical, biological, radiological, and nuclear (CBRN) incidents that result in surges of patient presentations. Chemical, biological, radiological, and nuclear in
Show more

40
Being a nurse during an earthquake that affected ten provinces: A qualitative study on experiences and expectations
Kaya, SS and Erdogan, EG
Mar 2025
INTERNATIONAL NURSING REVIEW
72 (1)
Enriched Cited References
AimThis study was conducted to determine the professional experiences and future expectations of nurses working in the most damaged areas during the first two weeks of the 2023 Turkey earthquake.BackgroundThe increase in the frequency and severity of disasters in recent years has strongly shown that nurses must be prepared to respond to all disasters. To prepare for disasters that require a mul
Show more

41
The Experiences of Nurses as First Responders to Disaster: A Qualitative Study
Açik, C and Sevim, TY

Jun 2025
NURSING & HEALTH SCIENCES
27 (2)
Enriched Cited References
This study aimed to explore the experiences of nurses who were the first to respond to the disaster, using a descriptive qualitative design. The sample consisted of 22 nurses who were nurses in the earthquake-affected provinces of Turkey or who were on assignment in the region after the disaster. Data were collected through individual in-depth interviews and analyzed using content analysis. The
Show more

42
Deployed in Disaster: Perspectives of Personnel Deployed in Ontario Long-Term Care During the Pandemic
Oldenburger, D; Baumann, A; (...); Baba, V
Sep 2025
INTERNATIONAL NURSING REVIEW
72 (3)
Enriched Cited References
Aim The aim of the study is to explore the experience and perspectives of healthcare personnel deployed from the hospitals and military into long-term care (LTC) in Ontario during the pandemic.Background Personnel from acute care hospitals, community care, the Canadian Armed Forces (CAF), and the Canadian Red Cross were deployed in support of LTC homes across Ontario.Introduction This article r
Show more

43
Experiences of adolescents affected by earthquakes: A qualitative study
Baskale, H and Solmaz, P
Jul 2024
JOURNAL FOR SPECIALISTS IN PEDIATRIC NURSING
29 (3)
Enriched Cited References
PurposeThe aim of the study is to explore the experiences of adolescents affected by the earthquake.Design and MethodsThis study was carried out in a descriptive phenomenological design, which is a qualitative research method. This research was conducted as a qualitative investigation utilizing a phenomenological approach to explore the experiences of 12 adolescents through the purposeful snowb
Show more

44
Challenges and Resources for Nurses Participating in a Hurricane Sandy Hospital Evacuation
Semantic search result
VanDevanter, N; Raveis, VH; (...); Keller, R
Nov 2017
JOURNAL OF NURSING SCHOLARSHIP
49 (6) , pp.635-643
PurposeWeather-related disasters have increased dramatically in recent years. In 2012, severe flooding as a result of Hurricane Sandy necessitated the mid-storm patient evacuation of New York University Langone Medical Center. The purpose of this study was to explore, from the nurses' perspective, what the challenges and resources were to carrying out their responsibilities, and what the implic
Show more

45
Cross-sectional survey of the disaster preparedness of nurses across the Asia-Pacific region
Semantic search result
Usher, K; Mills, J; (...); Woods, C
Dec 2015
NURSING & HEALTH SCIENCES
17 (4) , pp.434-443
Healthcare workers who have received disaster preparedness education are more likely to report a greater understanding of disaster preparedness. However, research indicates that current nursing curricula do not adequately prepare nurses to respond to disasters. This is the first study to assess Asia-Pacific nurses' perspectives about their level of disaster knowledge, skills, and preparedness. A

Show more

46
Learning from critical care nurses' wartime experiences and their long-term impacts
Segev, R
Mar 2023
NURSING IN CRITICAL CARE
28 (2) , pp.253-260
Enriched Cited References
Background The history of critical care nursing is intertwined with that of battlefield nursing, where for almost 200 years, nurses worked to save injured soldiers' lives, risking their own physical and emotional injuries. Today, with nurses increasingly deployed to provide critical care during natural, man-made and public health crises that can resemble battlefield situations, there is much to
Show more

47
Emergency Preparedness Competencies Among Nurses Implications for Nurse Administrators
Semantic search result
McNeill, C; Adams, L; (...); Alfred, D
Jul-aug 2020
JOURNAL OF NURSING ADMINISTRATION
50 (7-8) , pp.407-413
OBJECTIVE The aim of this study was to examine current levels of self-reported professional emergency preparedness competence among nurses. In addition, relationships between nurse professional emergency preparedness competence, personal preparation for a disaster, and perceived likelihood of reporting to work after a disaster are examined. BACKGROUND Evidence suggests wide gaps in nurses' fami
Show more

48
Building capacity in times of crisis: Increasing cultural competence of healthcare professionals in the context of the COVID-19
Slobodin, O; Kula, Y; (...); Cohen, O
Jun 2021 (Early Access)
JOURNAL OF CLINICAL NURSING
Background Nurses are the frontline healthcare professionals fighting the medical and social effects of the current COVID-19 pandemic. Although they work with diverse populations, there is a lack of literature on culturally competent education during an emergency such as a pandemic.
Aims and objectives To examine the effectiveness of an online education programme aimed at increasing cultu
Show more

49
The effect of disaster management training program on emergency nurses' knowledge, skills, and personal preparedness in Palestine
Semantic search result
Sa'd, RI and Malak, MZ
Jun 2025
INTERNATIONAL EMERGENCY NURSING
80
Purpose: Healthcare professionals including nurses have a crucial role in reducing the impact of severe crises, also emergency nurses are the majority of healthcare professionals who directly work with catastrophe victims. Thus, they should be prepared well to cope with such situations and they need to have appropriate knowledge, skills, and competencies to manage their tasks during disasters.
Show more

50
Investigating perceived core disaster competencies of nurses in Iran: A case study of northwest hospitals
Ziapour, A; Darabi, F; (...); Kianipour, N
Sep 2024
PUBLIC HEALTH NURSING
41 (5) , pp.970-978

Enriched Cited References
AimThis study was carried out to investigate the perception of the main competencies of disaster management in Iranian emergency department nurses in 2023 in hospitals affiliated with Kermanshah University of Medical Sciences. The present study was conducted to explore the perceived core disaster competencies in nurses of hospitals affiliated with Kermanshah University of Medical Sciences in 20
Show more

Improving Nurses' Disaster Skills: A Policy Brief on Perceived Core Disaster Competencies
Semantic search result
Ziapour, A and Darabi, F
Jul 2025
PUBLIC HEALTH NURSING
42 (4) , pp.1520-1524
Enriched Cited References
In the past two decades, disasters have directly affected the lives of nearly 1.23 million people. Asia is the most affected region in the world by all types of disasters, such as war and natural disasters, accounting for 44% of all disasters, 58% of total deaths, and 70% of all victims. Iran has remained one of the most heavily impacted countries by various disasters. Emergency nurses are expe
Show more

52
Disaster preparedness and core competencies among emergency nurses: A cross-sectional study
Semantic search result
Chegini, Z; Arab-Zozani, M; (...); Karkan, HA
Mar 2022
NURSING OPEN
9 (2) , pp.1294-1302
Enriched Cited References
Aim: With the rise in frequency and severity of disasters in recent decades, it is essentially important that nurses must be adequately prepared to handle them. This study was aimed to evaluate the levels of disaster core competencies and preparedness of nurses in the emergency department.
Design: A cross-sectional survey design was used.
Methods: This cross-sectional research was c
Show more

53
Experiences of Turkish nurses volunteering in the disaster zone following the 2023 Türkiye-Syria earthquake
Annak, IM; Erdogan, B and Emre, NY
Sep 2025
INTERNATIONAL NURSING REVIEW
72 (3)
Enriched Cited References
AimThe study aimed to determine the experiences of Turkish nurses who volunteered in the disaster zone following the 2023 T & uuml;rkiye-Syria earthquake.BackgroundThe earthquake devastated 11 cities in T & uuml;rkiye, which required a comprehensive humanitarian response. Despite the crucial role of nurses in crisis management, limited research exists on their experiences in earthquake zones.In
Show more

54
Assessing the frontline competency gap: Emergency care perceptions among nurses in Yemen's conflict zone
Mani, ZA; Innab, A and Taleb, F
Jun 2025
INTERNATIONAL NURSING REVIEW
72 (2)
Enriched Cited References
BackgroundNursing competencies in armed conflict situations are critical for effective response and recovery. This study explores nurses' perceptions regarding their competencies in armed conflict zones to identify areas of proficiency and those requiring further emphasis in training.MethodsThis cross-sectional descriptive study adhered to the STROBE reporting guidelines. It used a validated qu
Show more

55
Nursing skills required across natural and man-made disasters: A scoping review
Su, Y; Wu, XV; (...); Yang, Y
Oct 2022
JOURNAL OF ADVANCED NURSING
78 (10) , pp.3141-3158
Aims To map the nursing skills required for different types of disasters. Design This was a scoping review of research studies conducted between July and August 2021. We conducted a systematic literature search of nine electronic databases from inception till July 2021, and an updated search was done in April 2022. This review is based on the methodological framework of Arksey and O'Malley (200
Show more

56
Experiences of nurses involved in natural disaster relief: A meta-synthesis of qualitative literature
Xue, CL; Shu, YS; (...); Lee, A
Dec 2020
JOURNAL OF CLINICAL NURSING
29 (23-24) , pp.4514-4531
Enriched Cited References
Aim To explore nurses' experiences in natural disaster response. Background Nurses are key to disaster response. There is a growing body of qualitative research exploring this emerging nursing issue. However, there is a need to synthesise and summarise this body of knowledge to identify the overarching elements of how nurses experience working in disaster situations to reflect on their experien
Show more

57
Nurses' disaster preparedness and core competencies in Turkey: a descriptive correlational design
Taskiran, G and Baykal, U
Jun 2019
INTERNATIONAL NURSING REVIEW
66 (2) , pp.165-175
Aim This descriptive correlational study aimed to identify nurses' perceptions of their own disaster preparedness and core competencies. Background As disasters have increased in number and severity in recent years, it is crucial that nurses should be appropriately prepared. There is still limited research on this issue in Turkey. Introduction With changes in disaster policies in the last decad
Show more

58
Perceptions of knowledge of disaster management among military and civilian nurses in Saudi Arabia
Semantic search result
Al Thobaity, A; Plummer, V; (...); Copnell, B
Aug 2015
AUSTRALASIAN EMERGENCY NURSING JOURNAL 18 (3) , pp.156-164
Background: It is generally accepted that nurses have insufficient knowledge about disaster preparedness due to a lack of acceptance of core competencies and the absence of disaster preparedness in nursing curricula.(1) This study explored nurses' knowledge and sources of knowledge, and skills as they relate to disaster management in Saudi Arabia, where more than 4660 people have died, 32,000 p
Show more

59
A Qualitative Study of Disaster Resilience of Chinese Adolescents Five Years after Super Typhoon Rammasun
Yan, YN; Turale, S; (...); Klunklin, A
Apr-jun 2023
PACIFIC RIM INTERNATIONAL JOURNAL OF NURSING RESEARCH
27 (2) , pp.213-229
Adolescents and younger children are more vulnerable during and after disasters than adults, and their ability to develop resilience depends on many factors, including culture and context. However, adolescents' experiences and voices need to be considered more often in disaster research, since their understanding of disaster resilience is not known in depth. This study aimed to understand Chine

Show more

60

DISASTER KNOWLEDGE, SKILLS, AND PREPAREDNESS AMONG NURSES IN BENGKULU, INDONESIA: A DESCRIPTIVE CORRELATIONAL SURVEY STUDY

Semantic search result

Setyawati, AD; Lu, YY; (...); Liang, SY

Sep 2020

JOURNAL OF EMERGENCY NURSING

46 (5) , pp.633-641

Introduction: Registered nurses have to adequately prepare to respond to disasters, as they have become increasingly frequent worldwide. The aim of this study was to identify the factors influencing registered nurses' disaster preparedness in Bengkulu, Indonesia.

Methods: This study used a descriptive correlational study design. A total of 130 registered nurses working in 2 governmental h

Show more

61

Indonesian emergency nurses' preparedness to respond to disaster: A descriptive survey

Semantic search result

Rizqillah, AF and Suna, J

May 2018

AUSTRALASIAN EMERGENCY CARE

21 (2) , pp.64-68

Background: As frontline hospital staff, emergency nurses must be prepared to respond in disaster situations. While many disasters have befallen Indonesia, no available studies document disaster preparedness of nurses in this region. This study aimed to assess disaster preparedness among Indonesian emergency nurses, as well as examine factors that affected disaster preparedness in this group.</

Show more

62

Analysis of the effects of high-fidelity simulation on nursing students' perceptions of their preparedness for disasters

Semantic search result

Unver, V; Basak, T; (...); Tosune, N

May 2018

INTERNATIONAL EMERGENCY NURSING

38 , pp.3-9

Introduction: As disasters may occur any time, health care staff and institutions should be prepared to manage these events. The aim of study is to analyze the effects of high-fidelity simulation on the perceptions of senior nursing students regarding their preparedness for disasters.

Methods: This study used a pretest-posttest design and was conducted as a quasi-experimental investigatio

Show more

63

Disaster management: Emergency nursing and medical personnel's knowledge, attitude and practices of the East Coast region hospitals of Malaysia

Semantic search result

Ahayalimudin, N and Osman, NNS

Nov 2016

AUSTRALASIAN EMERGENCY NURSING JOURNAL 19 (4) , pp.203-209

Background: Disaster management is critical, as its insight could diminish the impact of a disaster, and participation of emergency medical personnel is crucial. This study explores emergency medical personnel's knowledge, attitude and practice towards disaster management.

Methodology: This study utilised a cross-sectional study design, and the data collected from 194 emergency nursing an

Show more

64

Hospital Nurses' Willingness to Respond in a Disaster

Semantic search result
Choi, HS and Lee, JE
Feb 2021
JOURNAL OF NURSING ADMINISTRATION
51 (2) , pp.81-88
OBJECTIVE
This study examined the factors associated with nurses' willingness to respond in a disaster. BACKGROUND Nurses are key personnel in case of disasters, and therefore, understanding factors associated with willingness to respond is important. METHODS
Questionnaires were distributed to 200 nurses recruited from 2 public hospitals in Seoul, Korea. Data were collected in
Show more

65
Nurses working in healthcare facilities during natural disasters: a qualitative enquiry
Semantic search result
Scrymgeour, GC; Smith, L; (...); Paton, D
Sep 2020
INTERNATIONAL NURSING REVIEW
67 (3) , pp.427-435
Aim To explore the ability of nurses to be adequately ready for and to respond to a disaster caused by a natural hazard. Background During a disaster involving a healthcare facility, nurses are commonly the largest group of healthcare workers impacted. The range of problems facing nurses working in healthcare facilities in Australia and New Zealand at the time of disasters triggered by earthqua
Show more

66
Educational needs and disaster response readiness: A cross-sectional study of clinical nurses
Semantic search result
Jang, I; Kim, JS; (...); Seo, Y
Jan 2021
JOURNAL OF ADVANCED NURSING
77 (1) , pp.189-197
Enriched Cited References
Aim To analyse clinical nurses' educational needs and disaster response readiness and the factors influencing their disaster response readiness. Design This was a cross-sectional study. Methods A convenience sample comprising 260 nurses with more than a year of working experience at a hospital in Korea was selected from 1-31 August 2019. Data on nurses' educational needs and disaster response r
Show more

67
Nurses' stories from Beirut: The 2020 explosive disaster on top of a pandemic and economic crises
Jabbour, R; Harakeh, M; (...); Darwish, H
Mar 2021
INTERNATIONAL NURSING REVIEW
68 (1) , pp.1-8
The World Health Organization designated last year as the International Year of the Nurse and the Midwife. And as we know worldwide, 2020 became an unforgettable year as nurses and midwives everywhere confronted the COVID-19 pandemic. To be a nurse in 2020 was challenging and heroic, but being a nurse in 2020 in Beirut, Lebanon was so extraordinarily charged with adversity. The country witnesse
Show more

68
Perceptions of disaster management knowledge and skills among advanced practice registered nurses
Semantic search result
Fil, SL; Champion, JD and Christiansen, B
Jul 2021
JOURNAL OF THE AMERICAN ASSOCIATION OF NURSE PRACTITIONERS
33 (7) , pp.514-520
Enriched Cited References

Background and purpose: Disaster management experts around the world agree that nurses play a vital role in disaster preparation, response, and recovery. The Joint Commission on Accreditation of Health care Organizations requires biannual disaster drills for accredited facilities, and there are numerous training resources and educational materials on disaster management topics. Multiple federal
Show more

69
Disaster education and preparedness in the acute care setting: A cross sectional survey of operating theatre nurse's disaster knowledge and education
Semantic search result
Sonneborn, O; Miller, C; (...); Cross, R
Jun 2018
NURSE EDUCATION TODAY
65 , pp.23-29
Background: Operating theatre services can be heavily relied upon during mass casualty disaster events, which require nurses to have adequate training and education of hospital disaster management plans to respond appropriately. The evidence-base of disaster preparedness in the acute setting is limited, particularly with regard to operating theatre nurses.
Objectives: Explore operating th
Show more

70
Why a disaster is not just normal business ramped up: Disaster response among ED nurses
Semantic search result
Hammad, KS; Arbon, P; (...); Hutton, A
Feb 2018
AUSTRALASIAN EMERGENCY CARE
21 (1) , pp.36-41
Background: The emergency department (ED) is a familiar place for the emergency nurse who spends their working days inside it. A disaster threatens that familiarity and creates changes that make working in the ED during a disaster response different from the everyday experience of working in the ED.
Methods: This research reports on an aspect of the findings from a larger study about the
Show more

71
Flood disaster preparedness experiences of hospital personnel in Thailand: A qualitative study
Rattanakanlaya, K; Sukonthasarn, A; (...); Chanprasit, C
Aug 2018
AUSTRALASIAN EMERGENCY CARE
21 (3) , pp.87-92
Background: Nurses, as well as other health personnel and health systems, worldwide need to be adequately prepared for disasters because it is often difficult to predict where and when disasters strike. The 2011 Thailand flood disaster caused significant damage, including to hospitals. The purpose of this study was to investigate the experiences of hospital personnel regarding flood disaster pr
Show more

72
Nursing Education for Disaster Preparedness and Response
Semantic search result
Wilkinson, AM and Matzo, M
Feb 2015
JOURNAL OF CONTINUING EDUCATION IN NURSING
46 (2) , pp.65-73
Catastrophic mass casualty events (MCEs), such as pandemic influenza outbreaks, earthquakes, or large-scale terrorism-related events, quickly and suddenly yield thousands of victims whose needs overwhelm local and regional health care systems, personnel, and resources. Such conditions require deploying scarce resources in a manner that is different from the more common multiple casualty event.
Show more

73

Disaster-Related Community Resilience: A Concept Analysis and a Call to Action for Nurses
Semantic search result
Heagele, T
May-jun 2017
PUBLIC HEALTH NURSING
34 (3) , pp.295-302
A paucity of nursing literature is available on disaster-related community resilience. Using a nursing method for analyzing concepts, this article attempts to clarify the meaning of this novel concept to encourage nursing research and practice. This concept analysis provides an introduction to the phenomenon of disaster-related community resilience for nurses and consumers of nursing research.
Show more

74
Exploring nurse vs. NP disaster response competencies
Semantic search result
Adelman, DS; Fant, C; (...); Zak, C
Dec 2019
NURSE PRACTITIONER
44 (12) , pp.42-48
This second installment of the Disaster Response Series covers the nurse competencies for disaster response outlined by the International Council of Nurses and the World Health Organization and explores how NP disaster response competencies from the National Organization of Nurse Practitioner Faculties align with nurse competencies.

75
National Disaster Health Consortium Competency-Based Training and a Report on the American Nurses Credentialing Center Disaster Certification Development
Semantic search result
Smith, SJ and Farra, SL
Dec 2016
NURSING CLINICS OF NORTH AMERICA
51 (4) , pp.555-+
As the largest profession of health care providers, nurses are an integral component of disaster response. Having clearly delineated competencies and developing training to acquire those competencies are needed to ensure nurses are ready when disasters occur. This article provides a review of nursing and interprofessional disaster competencies and development of a new interprofessional disaster
Show more

CINAHL
N= 82, included 0, duplicates 34, removed 48

Major disaster, profound impact: A qualitative examination of emergency department nurses' experiences during the 2023 Turkey Maraş earthquake.

Scientific response to the 2023 Kahramanmaraş earthquake: A bibliometric study.

Unveiling the heart of disaster nursing: A qualitative study on motivations, challenges, and lessons from the devastating 2023 Turkey earthquakes.

Risk Mitigation Measures Captured by a Tertiary Hospital's Disaster Simulation: An Observational Study.

Nursing as an earthquake survivor: A qualitative study on nurses traumatized by the Kahramanmaraş earthquake in Turkey.

Disaster Nursing Competencies in a Time of Global Conflicts and Climate Crises: A Cross-Sectional Survey Study.

Experiences and psychosocial challenges of volunteer nurses in Turkey devastating earthquake zones: Lessons to be learnt for prevention of health system problems in disasters: A qualitative study.

Investigating perceived core disaster competencies of nurses in Iran: A case study of northwest hospitals.

Triage ethics in mass casualty incident simulation: A phenomenological exploration.

Overcoming challenges in nursing disaster preparedness and response: an umbrella review.

Evaluating nurses' psychological and operational preparedness for mass-casualty events in Saudi Arabia.

Disaster nursing and disaster preparedness: an investigation of nursing students' knowledge, competence and attitudes.

Identifying the Challenges of Prehospital and Hospital Emergency Services During the Management of Alcohol Poisoning Disaster in the City of Rafsanjan.

Evaluation of prehospital preparedness for major incidents on a national level, with focus on mass casualty incidents.

Disaster preparedness of health professionals at mass gatherings: a scoping review.

Managing multiple-casualty incidents: a rural medical preparedness training assessment.

Helpful lessons from the didactic concepts of shore-based triage exercises for preparing first responders for mass casualty incidents on board cruise ships...17th International Symposium on Maritime Health, June 11-14, 2025, Rotterdam, Netherlands.

Kuala Lumpur train collision during the COVID-19 pandemic.

Why a disaster is not just normal business ramped up: Disaster response among ED nurses.

Evaluation of the decision-making process within the table-top exercise of the Terror and Disaster Surgical Care (TDSC®) course.

Pre-hospital emergency care personnel's challenges in providing care in mass casualty incidents: A qualitative study.

Evaluation of pediatric gunshot wounds and emergency department dynamics in high-volume incidents.

Trends and ethical issues in nursing during disasters: A systematic review.

Improvements in State and Local Planning for Mass Dispensing of Medical Countermeasures: The Technical Assistance Review Program, United States, 2007–2014.

The psychological impact of disaster on first responders in the aftermath of Bahanaga train tragedy: A mixed methods approach.

A Qualitative Study on Researchers' Experiences after Publishing Scientific Reports on Major Incidents, Mass-Casualty Incidents, and Disasters.

Recommendations for burns care in mass casualty incidents: WHO Emergency Medical Teams Technical Working Group on Burns (WHO TWGB) 2017-2020.

Full-scale regional exercises: Closing the gaps in disaster preparedness.

Facilitators and constrainers of civilian–military collaboration: the Swedish perspectives.

Assessment of the Capacity and Capability of Burn Centers to Respond to Burn Disasters in Belgium: A Mixed-Method Study.

Organizing Health Care Services for the 2017 "Athens Marathon, The Authentic:" Perspectives on Collaboration among Health and Safety Personnel in the Marathon Command Center.

The aftermath of the Kuwait mosque bombing: A retrospective cohort analysis and lessons learned.

Developing a Mass Casualty Surge Capacity Protocol for Emergency Medical Services to Use for Patient Distribution.

Kuala Lumpur train collision during the COVID-19 pandemic.

Rescue workers had greater confidence in their own mastery after the terror attacks of 22 July 2011.

Challenges in mass fatality management: A case study of the 2010 Haiti earthquake.

Self-reported Preparedness to Respond to Mass Fatality Incidents in 38 State Health Departments.

Experiences of adolescents affected by earthquakes: A qualitative study.

Recommendations for Improving Stop the Bleed: A Systematic Review.

Barriers and challenges to multiple-casualty management systems: a single-centre, cross-sectional study at the Nasser Medical Complex, Gaza Strip.

Hospital Administration and Nursing Leadership in Disasters.

Challenges of Burn Mass Casualty Incidents in the Prehospital Setting: Lessons From the Formosa Fun Coast Park Color Party.

Kabul airport suicide bombing attack: Mass casualty management at the EMERGENCY NGO Hospital.

Developing a National Trauma Research Action Plan: Results from the prehospital and mass casualty research Delphi survey.

The psychological experiences of Lebanese ground zero front-line nurses during the most recent COVID-19 outbreak post Beirut blast: A qualitative study.

Education, training and technological innovation, key components of the ESTES-NIGHTINGALE project cooperation for Mass Casualty Incident preparedness in Europe.

Feasibility of Telesimulation and Google Glass for Mass Casualty Triage Education and Training.

Allied Dental Students' DVI Learning Outcomes Following a Multimedia Module...American Dental Hygienists Association (ADHA) Annual Conference 2022, June 23-26, 2022, Louisville, Kentucky

UK healthcare staff experiences and perceptions of a mass casualty terrorist incident response: a mixed-methods study.

Between the devil and the deep blue sea: A review of 25 modern naval mass casualty incidents with implications for future Distributed Maritime Operations.

Evaluating an 'incident control' approach to non-communicable disease.

The Dermal Exposure Risk Management and Logic eToolkit: Characterizing and managing dermal exposure during emergency management operations.

The history of disaster nursing: from Nightingale to nursing in the 21st century.

Comparing virtual reality and live standardized patient drill simulation-based triage training methods in terms of triage knowledge and performance.

Development and Evaluation of an Undergraduate Training Course for Developing International Council of Nurses Disaster Nursing Competencies in China.

Moral Distress among Disaster Responders: What is it?

A dynamic mass casualty incident at sea: Lessons learned from the Mavi Marmara.

Bomb blast in a tertiary care hospital, the challenges faced during management of victims in a resource limited country.

Human Stampedes: An Updated Review of Current Literature.

Using Big Data to Study the Impact of Mass Violence: Opportunities for the Traumatic Stress Field.

The Challenges and Opportunities in Disaster Nursing Education in Turkey.

Chinese nurses' relief experiences following two earthquakes: Implications for disaster education and policy development.

Description of Patients Medications Needs and the Community Pharmacist's Role in Puerto Rico Following a Natural Disaster.

Work Stress, Resilience, and Professional Quality of Life Among Nurses Caring for Mass Burn Casualty Patients After Formosa Color Dust Explosion.

Volunteer nurses' learning experiences in Ludian County, Yunnan, China: Implication for public health nursing education in a disaster.

Disaster nursing in Iran: Challenges and opportunities.

Outcomes from two forms of training for first-responder competency in cholinergic crisis management.

Ethical and legal challenges associated with disaster nursing.

Multidisciplinary Team Response to Support Survivors of Mass Casualty Disasters: A Systematic Review Protocol.

Disaster nursing experiences of Chinese nurses responding to the Sichuan Ya'an earthquake.

Survey of trauma registry data on tourniquet use in pediatric war casualties.

Experience of Bangladeshi occupational therapists with "Rana Plaza Tragedy" survivors: recovery and rehabilitation phases of disaster management.

Online Victim Tracking and Tracing System (ViTTS) for Major Incident Casualties.

Disaster nursing skills, knowledge and attitudes required in earthquake relief: Implications for nursing education.

Challenges of major incident management when excess resources are allocated: experiences from a mass casualty incident after roof collapse of a military command center.

Educator's forum. Nursing students' perceptions about disaster nursing.

Nurses as Leaders in Disaster Preparedness and Response-A Call to Action.

Development of disaster nursing education and training programs in the past 20 years (2000–2019): A systematic review.

Chinese nurses' experience in the Wenchuan earthquake relief.

Use of 'shuttered' hospitals to expand surge capacity.

A recommended epidemiological study design for examining the adverse health effects among emergency workers who experienced the TEPCO fukushima daiichi NPP accident in 2011.

Death on the battlefield (2001-2011): Implications for the future of combat casualty care.
